# Suppression of Breast Tumor Growth and Metastasis by an Engineered Transcription Factor

**DOI:** 10.1371/journal.pone.0024595

**Published:** 2011-09-13

**Authors:** Adriana S. Beltran, Angela Russo, Haydee Lara, Cheng Fan, Paul M. Lizardi, Pilar Blancafort

**Affiliations:** 1 Department of Pharmacology, University of North Carolina at Chapel Hill, Chapel Hill, North Carolina, United States of America; 2 Lineberger Comprehensive Cancer Center, University of North Carolina at Chapel Hill, Chapel Hill, North Carolina, United States of America; 3 Department of Pharmacology, University of Illinois at Chicago, Chicago, Illinois, United States of America; 4 Department of Marine Biotechnology, CICESE Research Institute, Ensenada, Mexico; 5 Department of Pathology, Yale University School of Medicine, New Haven, Connecticut, United States of America; Baylor College of Medicine, United States of America

## Abstract

*Maspin* is a tumor and metastasis suppressor playing an essential role as gatekeeper of tumor progression. It is highly expressed in epithelial cells but is silenced in the onset of metastatic disease by epigenetic mechanisms. Reprogramming of *Maspin* epigenetic silencing offers a therapeutic potential to lock metastatic progression. Herein we have investigated the ability of the Artificial Transcription Factor 126 (ATF-126) designed to upregulate the *Maspin* promoter to inhibit tumor progression in pre-established breast tumors in immunodeficient mice. ATF-126 was transduced in the aggressive, mesenchymal-like and triple negative breast cancer line, MDA-MB-231. Induction of ATF expression *in vivo* by Doxycycline resulted in 50% reduction in tumor growth and totally abolished tumor cell colonization. Genome-wide transcriptional profiles of ATF-induced cells revealed a gene signature that was found over-represented in estrogen receptor positive (ER+) “Normal-like” intrinsic subtype of breast cancer and in poorly aggressive, ER+ luminal A breast cancer cell lines. The comparison transcriptional profiles of ATF-126 and *Maspin* cDNA defined an overlapping 19-gene signature, comprising novel targets downstream the *Maspin* signaling cascade. Our data suggest that *Maspin* up-regulates downstream tumor and metastasis suppressor genes that are silenced in breast cancers, and are normally expressed in the neural system, including *CARNS1*, *SLC8A2* and *DACT3*. In addition, ATF-126 and *Maspin* cDNA induction led to the re-activation of tumor suppressive miRNAs also expressed in neural cells, such as miR-1 and miR-34, and to the down-regulation of potential oncogenic miRNAs, such as miR-10b, miR-124, and miR-363. As expected from its over-representation in ER+ tumors, the ATF-126-gene signature predicted favorable prognosis for breast cancer patients. Our results describe for the first time an ATF able to reduce tumor growth and metastatic colonization by epigenetic reactivation of a dormant, normal-like, and more differentiated gene program.

## Introduction

*Mammary Serine Protease Inhibitor* (*Maspin*, *SERPINB5*) is a multifunctional protein possessing tumor and metastasis suppressive functions [1], [2]. Additionally, *Maspin* over-expression inhibits *in vivo* angiogenesis [3]. The multifaceted nature of *Maspin* affecting many molecular mechanisms during neoplastic disease progression makes it a very attractive target in cancer biology. Importantly, clinical data shows that high *Maspin* levels are associated with better prognosis in breast, lung and prostate carcinomas [4], [5], [6].

As a class II tumor suppressor gene, *Maspin* is not mutated, rearranged or deleted in tumor cells. Instead, its expression is regulated by means of transcription factors [7] and epigenetic modifiers [8], [9]. While *Maspin* is expressed at high levels by epithelial cells, it is down-regulated in mesenchymal cells, such as stromal fibroblasts. In breast cancer cell lines and cancer specimens, silencing of *Maspin* correlates with acquisition of invasive and metastatic behavior. Epigenetic mechanisms controlling *Maspin* silencing include both, DNA [9] and H3K9 histone methylation [10]. Hence epigenetic mechanisms are reversible yet inherited during cell division, blockade of *Maspin* promoter silencing offers a potent strategy to reactivate tumor suppressor function. To this end, we have previously described the construction of Artificial Transcription Factors (ATFs) made of sequence-specific six Zinc Finger (ZF) domains[11] designed to bind unique 18-base pair recognition sites in the *Maspin* proximal promoter [12]. The ZFs were linked to a VP64 transactivator domain, which mediates a strong promoter up-regulation by recruitment of the *pol*II transcriptional complex. In cell systems, both in lung and breast cancer cell lines, retroviral transduction of one of the ATFs, ATF-126, led to a potent induction of apoptosis and inhibition of cell invasion [12], [13]. Furthermore, these ATFs were able to directionally demethylate the *Maspin* promoter and this effect depended on upon the orientation of the ATF along the DNA [13]. Consistently, we found that ATFs synergized with both methyltransferase and histone deacetylase inhibitors to reactivate silenced *Maspin* [13], [14], [15].

These previous observations suggested that ATF-126 was able to partially reprogram or revert the epigenetic state of the *Maspin* promoter, resulting in a re-activation of the endogenous gene. However, the impact of ATF-126 in inhibiting tumor progression in preexisting tumors and/or metastases *in vivo* has never been addressed. Herein, we have taken advantage of an inducible viral vector system to control the expression of ATF-126 in pre-existing breast tumor growths and experimental metastases in immunodeficient mice. Chemical induction of ATF-126 *in vivo* resulted in tumor suppression as well as in inhibition of breast tumor cell colonization. Furthermore, genome-wide DNA microarrays of MDA-MB-231 cells induced with ATF-126 revealed that breast tumor cells acquired a 550-gene signature that was found over-represented in estrogen receptor positive (ER+) breast cancer cell lines and in the normal-like intrinsic subtype of breast cancer. Our data indicates that ATF-126 up-regulates novel *Maspin*-dependent targets possessing tumor and metastasis suppressive functions, which are found epigenetically silenced in aggressive tumors. Our results outline a possible mechanism by which ATF-126 reprograms aggressive tumor cells towards a more “normal-like”, more benign, and more differentiated “epithelial-like” phenotype.

## Results

### Induction of ATF-126 by DOX results in endogenous reactivation of *Maspin* in MDA-MB-231 breast cancer cells

In order to monitor the effect of ATF-126 in inhibiting tumor progression in pre-existing tumors, we cloned the ATF-126 gene into an inducible TetOn retroviral vector (**Fig. S1A**). In this expression system the ATF expression was activated only in presence of the chemical inducer, Doxycycline (DOX). The MDA-MB-231-LUC cell line stably engineered with a luciferase (LUC) gene was transduced with either a control (empty retroviral vector) or the same vector expressing ATF-126. The LUC gene allowed the non-invasive monitoring of tumor growth and dissemination in a mouse model, using bioluminescence imaging, BLI (**Fig. S1B**).

**Figure 1:**
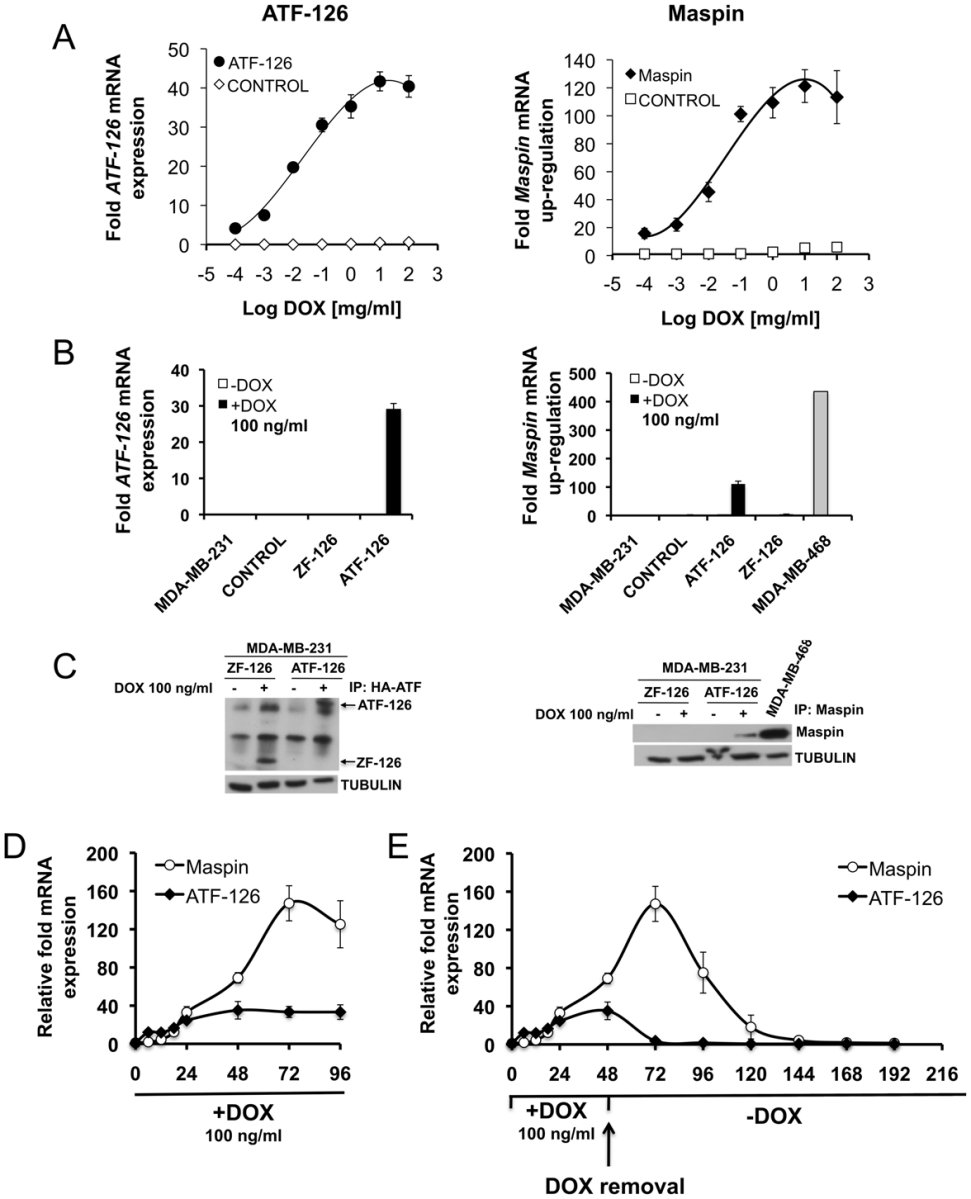
Induction of ATF-126 by DOX results in reactivation of the target gene *Maspin*. **A.** Dose-response plot monitoring *ATF-126* (left panel) and *Maspin* (right panel) mRNA levels upon treatment with increasing concentrations of DOX. CONTROL and ATF-126 cells were treated for 72 hours and mRNA was measured by quantitative real-time PCR (qRT-PCR). **B–C.** *ATF-126* and *Maspin* mRNA expression levels by qRT-PCR (B) and western blot (C) induced with 100 ng/ml of DOX. MDA-MB-468 is a poorly aggressive ER- breast cancer cell line expressing endogenous *Maspin* as a reference control [12]. **D.** Time course kinetics *of ATF-126* and *Maspin* mRNA levels by qRT-PCR upon DOX treatment. ATF-126 cells were induced with DOX and collected at 0, 6, 12, 18, 24, 48, 72 and 96 hours. **E.** Time course kinetics *of ATF-126* and *Maspin* expression levels by qRT-PCR upon DOX treatment and removal. ATF-126 cells were induced with DOX for 48 hours, then DOX was removed from the media and cells were maintained in DOX-free media for an additional 168 hours. Gene expression levels were normalized to the −DOX cells. Data represents the mean ± SD of three independent biological replicates. MDA-MB-231-LUC are un-transduced cells; CONTROL, cells transduced with an empty vector; ATF-126, a full length ATF containing the 6 ZF DNA-binding domains and VP64 activator domain; ZF-126, a truncated or inactive ATF-126 lacking the VP64 activator domain.

The effects of inducing ATF-126 with DOX were first monitored in cell culture assays. As shown in **Fig. 1A**, induction of the full length ATF-126 (comprising the specific 6ZF DNA-binding domains and the VP64 transactivator domain) resulted in a dose-dependent ATF-126 expression, as assessed by qRT-PCR. The induction of the ATF was accompanied by a concomitant up-regulation of the *Maspin* target (**Fig. 1B**). For subsequent studies we used a concentration of DOX of 100 ng/ml at which the expression of both, ATF-126 and *Maspin*, reached saturation.

Efficient ATF-126 induction was also verified by immunoprecipitation (using an anti-HA antibody recognizing the C-terminal HA epitope of the ATF; **Fig. 1C**, left panel). *Maspin* up-regulation in +DOX cells was also verified by immunoprecipitation (**Fig. 1C**, right panel). As shown in **Fig. 1D**, ATF-126 +DOX cells up-regulated *Maspin* quickly after ATF-126 expression (6–12 hours after addition of DOX), as expected from direct transcriptional regulation. A saturation of ATF expression was reached 48 hours after the addition of the drug, whereas that *Maspin* up-regulation achieved a maximum at 72 hours post-induction (**Fig. 1D**). Removal of DOX from the cell culture of ATF-126 cells also resulted in a decay of ATF expression that was evident 24 hours upon the retrieval of the drug. However, 72 hours after DOX removal, substantial *Maspin* expression was still detected in the cells even though ATF-126 mRNA expression was not. These results suggested that ATF-126 was able to transiently impact the epigenetic and transcriptional status of the *Maspin* promoter. Previously we have reported that ATF-126 was able to demethylate the *Maspin* proximal promoter [13]. Moreover, It is possible that the endogenous mechanisms responsible for *Maspin* silencing (including endogenous DNA-methylation processes) would be restored 96 hours after removal of DOX in ATF-126 transduced cells.

### ATF up-regulation by DOX results in *Maspin*-dependent induction of apoptosis

**Figure 2:**
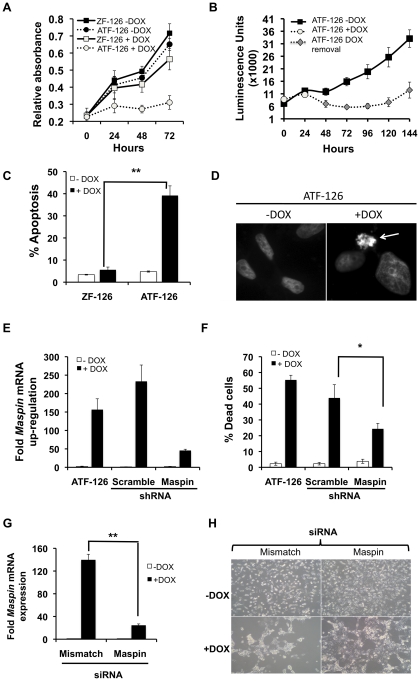
ATF-126 induced apoptosis upon DOX treatment. **A.** Cell viability plot of ATF-126 and ZF-126 cells treated with vehicle (−DOX) or DOX (+DOX) for 72 hours. ATF-126 refers to a full length, active ATF and ZF-126 is a truncated, transcriptionally inactive construct lacking the VP64 activator domain. **B.** Cell viability plot of ATF-126 transduced cells after DOX removal. Cells were induced with DOX for 48 hours (open circles). DOX was removed from the media and cells were kept in DOX-free media for an additional period of 96 hours. Viability of ATF-126 cells growing in absence of DOX (−DOX, filled squares) was plotted as reference control. Cell viability was measured with an XTT assay [12]. **C.** ATF-126 induced apoptosis upon DOX treatment. Either ATF-126 or ZF-126 cells were kept in vehicle-treated media (−DOX) or DOX (+DOX) and collected at 72 hours after induction. The percentage of apoptosis was quantitatively analyzed using an Annexin-V staining [12]. **D.** Hoechst staining of ATF-126 −DOX and +DOX cells at 72 hours post-induction. Arrow points to a positive apoptotic cell, showing nuclear condensation. **E.** Expression of *Maspin* by qRT-PCR in ATF-126 cells retrovirally transduced with either a scramble or with a *Maspin*-specific shRNA construct. **F.** Percentage of cell death by trypan-blue exclusion assay in ATF-126 cells transduced with either a scramble or a *Maspin*-specific shRNA. **G.** *Maspin* expression as assessed by qRT-PCR in ATF-126 cells transfected with either a mismatch or a *Maspin*-specific siRNA, and treated with vehicle (−DOX) or DOX (+DOX). **H.** Representative pictures of the *Maspin* siRNA knock-down experiments of cells treated with vehicle (−DOX) or DOX (+DOX). Bar graphs in C, E, F and G represent the average of 3 independent experiments. Differences between samples were calculated with a student t test with level of significance *p≤0.05 and ** p≤0.01.

Endogenous expression of tumor suppressors, including *Maspin*, has been associated with reduction of tumor cell viability by induction of apoptosis [12], [16]. Next, we studied if ATF-126 induction resulted in a reduction of tumor cell growth. As shown in **Fig. 2A**, the reactivation of ATF-126 (ATF-126 +DOX) led to 70% reduction in tumor cell viability relative to the same cells in absence of DOX (ATF-126 −DOX). As expected, no difference in cell viability was observed between CONTROL and ZF-126 (inactive ATF-126 lacking the activator domain) cells upon DOX induction. Interestingly, removal of DOX in ATF-126 cells led to a maintenance of the cell proliferation defect for 96 hrs after DOX removal (**Fig. 2B**), and approximately over four cell generations. This maintenance of tumor suppression correlates with the time window of *Maspin* transcriptional activation of **Fig. 1E**. In addition to cell proliferation defects, ATF-126 +DOX cells exhibited a 40% induction of apoptosis, as assessed by Annexin-V staining, which monitors early apoptosis (**Fig. 2C**). Similarly, 30–40% of ATF-126 +DOX cells were positive for the Hoechst staining, which labels apoptotic nuclei (**Fig. 2D**).

To verify that the apoptotic response triggered by ATF-126 was due to *Maspin* reactivation, we challenged ATF-126 cells with either a *Maspin*-specific or scramble shRNA constructs. The *Maspin*-shRNA in ATF-126 +DOX cells led to a 60% reduction of *Maspin* mRNA expression (**Fig. 2E**). As expected, a scramble shRNA construct did not significantly impact *Maspin* expression. Consistently, the scramble-shRNA in ATF-126 +DOX cells induced similar levels of cell death relative to the original ATF-126 +DOX cell line (**Fig. 2F**). However, the *Maspin*-shRNA in ATF-126 +DOX cells was able to rescue the phenotype, and a significant reduction of cell death (55% of reduction) was observed in the *Maspin* knock-down relative to the scramble and the ATF-126 parental line +DOX (**Fig. 2F**). Although the rescue of the cell death phenotype was not 100%, our results suggest that ATF-126 mediate reduction in tumor cell growth by primarily activation of its designed target *Maspin*. The incomplete rescue of the phenotype could be due the fact that the *Maspin* shRNA did not completely knocked down the mRNA transcript levels of *Maspin*, as shown in **Fig. 2E**. A siRNA approach further confirmed that the tumor suppressive phenotype observed in ATF-126 +DOX cells was due to *Maspin* re-activation. As shown in **Figs. 2G–H**, a *Maspin*-specific siRNA but not a non-specific mismatch (scramble) siRNA rescued the proliferation defect of ATF-126 +DOX cells. Overall these results support the conclusion that the cell death phenotype induced by ATF-126 was dependent on the *Maspin* target.

### ATF-126 reduces xenograft tumor growth and suppresses metastatic colonization of MDA-MB-231 cells upon DOX induction in immunodeficient mice

**Figure 3:**
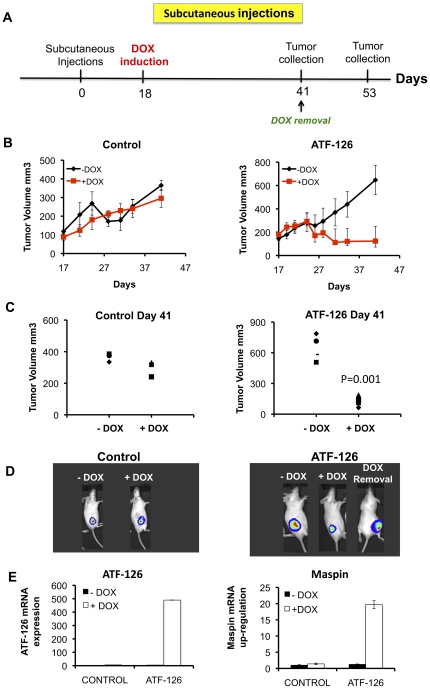
ATF-126 induced tumor suppression in SCID mice. **A.** Time-line of the experiments involving subcutaneous tumor injections illustrating the time of injection of tumor cells, induction and removal of DOX, and tumor collection. **B.** Time course plots monitoring tumor volumes of CONTROL and ATF-126 groups, N = 8 animals per group. Four animals per group were maintained in DOX-free diet (−DOX), whereas the other half was treated with DOX (+DOX). The tumor growth was measured by caliper the day before induction (day 17) until tumor collection (day 41). **C.** Tumor volume measurements at the day of tumor collection for both CONTROL (left panel) and ATF-126 animals (right panel). Differences in tumor growth were calculated with a student t test (***p = 0.001). **D.** Representative bioluminescence images comparing signal intensities of luciferase photon counts from the subcutaneous growths at day 41 for CONTROL (left) and ATF-126 animals (right), treated in absence (−DOX) and presence (+DOX) of DOX. “DOX removal” indicates an ATF-126-injected animal previously induced with DOX, subsequently removed from DOX (day 41), and imaged at day 53 (right). **E.** *ATF-126* and *Maspin* expression levels by qRT-PCR in tumor samples collected at day 41 from both, CONTROL and ATF-126 animals.

To investigate the ability of ATF-126 to reduce tumor cell growth in pre-existing breast tumor xenografts, we implanted either control 1×10^6^ MDA-MB-231-LUC cells or ATF-126 cells in SCID mice (N = 8 animals per group). Animals were kept in DOX-free conditions until tumors reached 100–200 mm^3^ (**Fig. 3A**). Eighteen days post-induction, half of the animals for control and ATF-126 groups were maintained in DOX-free diet, whereas the other half was switched to a +DOX diet. Tumor volume was assessed from the day before induction until tumor collection (day 41 post-injection), and BLI imaging was performed once a week. One of the animals belonging to the ATF-126 group previously induced with DOX was switched back to a DOX-free diet and sacrificed at day 53 post-injection (**Fig. 3A**). As shown in **Fig. 3B–C** (left panels), no statistical differences in tumor growth were observed between CONTROL +DOX and -DOX animals. In contrast, ATF-126 animals induced with DOX exhibited a significant reduction of tumor volume (approximately 50% reduction) that was stably maintained until the mice were sacrificed at day 41 (**Fig. 3B–C**, right panels).

Interestingly, induced ATF-126 animals that were removed from DOX at day 41 experienced a tumor relapse (**Fig. 3D**). This tumor recovery suggests that, like we have observed *in vitro*, long-term absence of ATF expression results in a re-establishment of *Maspin* silencing. Analysis of tumors recovered from the animals at day 41 demonstrated that the ATF-126 +DOX animals retained efficient ATF mRNA up-regulation (500-fold relative to un-induced animals). This induction of ATF mRNA was accompanied by a 20-fold *Maspin* up-regulation relative to −DOX animals (**Fig. 3E**). Overall these results demonstrate that ATF-126 was properly up-regulated in the tumor xenograft experiment and that this induction correlated with *Maspin* reactivation and with the maintenance of tumor suppressive functions.

**Figure 4:**
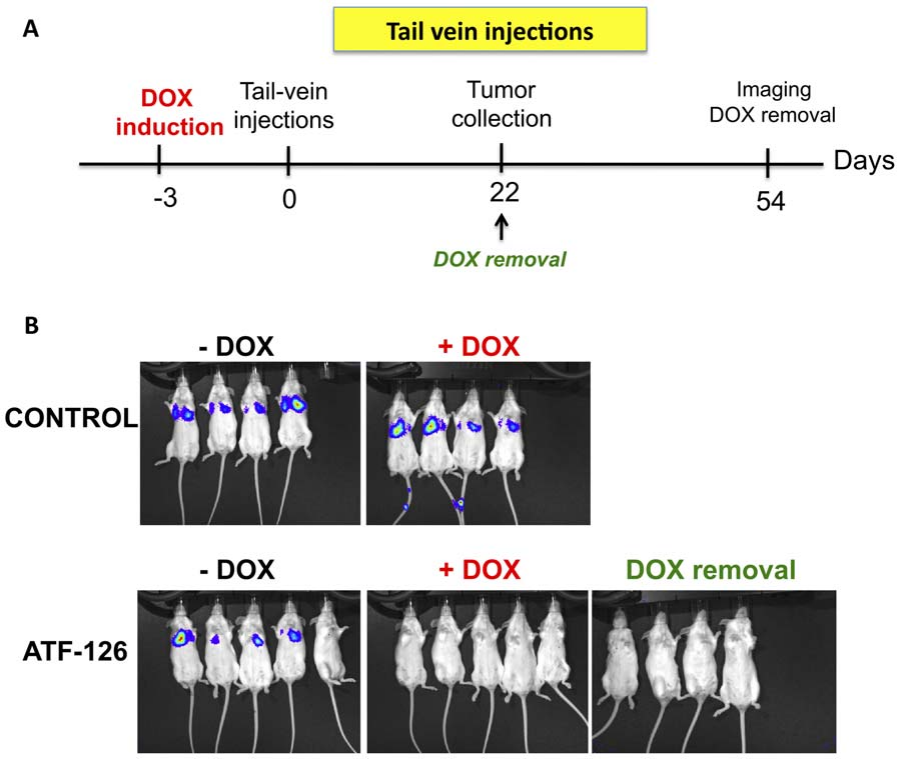
ATF-126 inhibits breast tumor cell colonization in the lungs. **A.** Time-line of the tail-vein injections experiments, indicating the times of the DOX induction and removal, and tumor collection. **B.** Bioluminescence images of CONTROL and ATF-126 mice groups maintained in DOX-free (−DOX, left panel) and DOX containing diet (+DOX, right panel). Mice were injected via tail-vein with either CONTROL or ATF-126 cells and imaged every week to assess lung colonization. Images shown were taken at day 22 after injection of the tumor cells. DOX removal indicates ATF-126 animals previously induced by DOX and next placed in DOX-free diet.

It is well documented for breast, prostate and lung carcinomas that high *Maspin* expression correlates with a less aggressive or metastatic behavior [17], [18], [19]. We next investigated the ability of ATF-126 to inhibit breast tumor colonization or experimental metastasis formation in immunodeficient mice. To address this, either 1×10^5^ MDA-MB-231-LUC CONTROL or ATF-126 cells were injected tail vein in SCID mice (N = 24). Twelve mice per group were maintained in DOX-free diet and 12 in +DOX diet. Because the need of the animals to adapt to a DOX diet, mice were feed three days prior to the injection (**Fig. 4A**). **Fig. 4B** (top panel) shows that CONTROL +DOX and −DOX cells effectively colonized the lungs and metastases were evident at day 14 post-injection. Similarly, the majority of the animals injected with ATF-126 maintained in DOX-free diet effectively colonized the lungs with similar signal intensities than CONTROLS. In contrast, ATF-126 animals in +DOX diet completely suppressed experimental metastatic colonization at day 21 (**Fig. 4B**, bottom panel). Moreover, removal of DOX from the diet of ATF-126 animals maintained these mice free of any detectable lung colonization even 54 days post-injection (**Fig. 4B**, bottom right panel). Overall these results indicated that ATF-126 effectively suppressed metastatic colonization. Whether the ATF-126 is equally effective in suppressing or reducing secondary tumor growth in well-established macrometastases will require further investigation.

### ATF-126 up-regulate a gene signature over-represented in the normal-like intrinsic subtype of breast cancer and ER+ cancer cell lines

The above results demonstrated that ATF-126 reduced tumor growth and suppressed metastatic colonization of the MDA-MB-231-LUC line. We next began the investigation of potential mechanisms by which ATF-126 could mediate its suppressive functions by performing genome-wide microarray analyses. CONTROL and ATF-126 cells where induced with DOX for 72 hours. Genes differentially regulated between −DOX and +DOX groups were determined by SAM analyses, with three independent arrays performed for each group. These analyses generated a robust 550-gene signature, defined as group of genes differentially up-regulated by ATF-126 (see **Table S1**). A Gene Ontology (GO) analysis revealed that multiple *Maspin* downstream pathways were reactivated by ATF-126, including tight junction and cell invasion, TGF-beta, and p53 signaling (**Table S2**).

**Figure 5:**
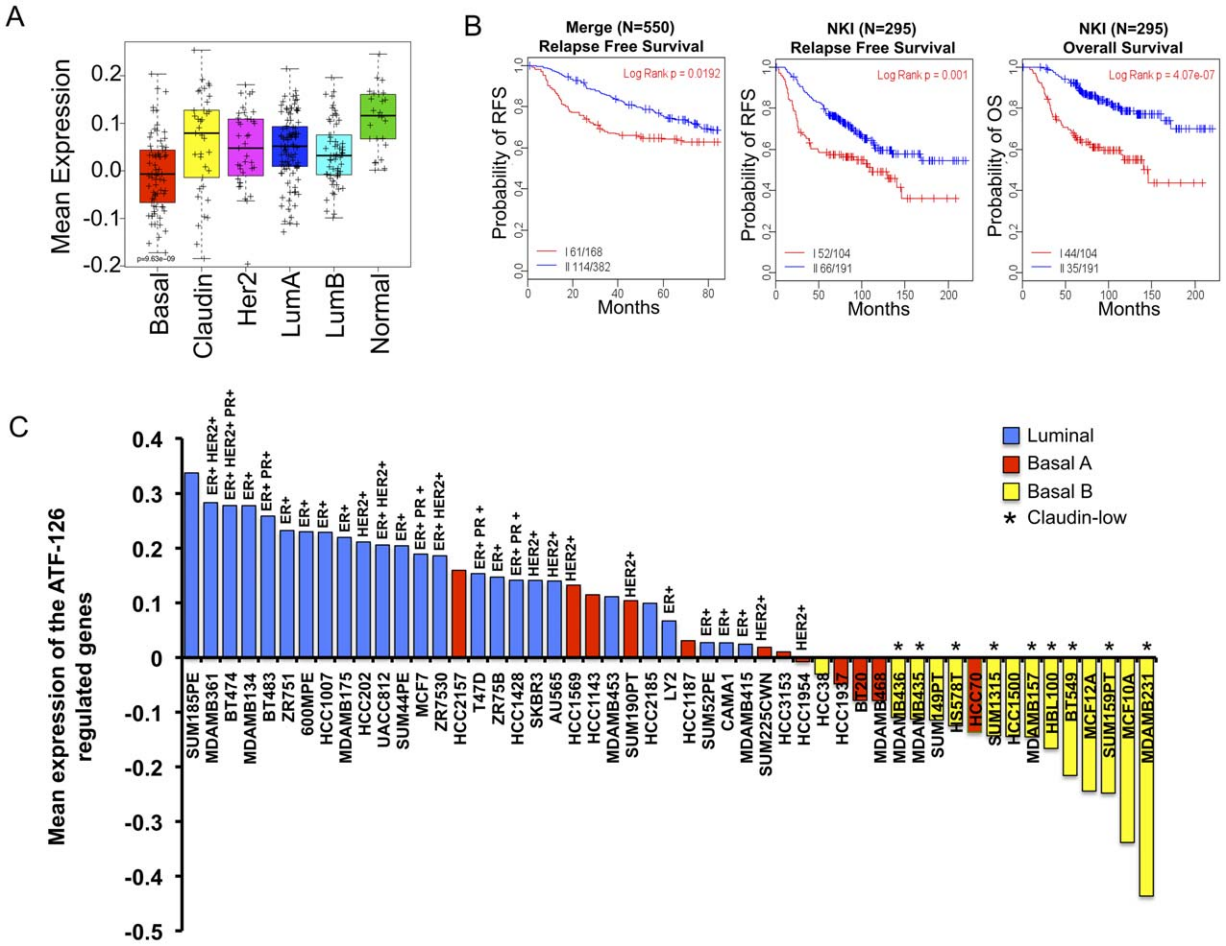
ATF-126 up-regulates a gene signature over-represented in Normal-Like breast cancers. **A.** Box-and-whisker plot for the mean expression of the 550 up-regulated gene signature (**Table S1**) This signature represents the number of genes significantly up-regulated in ATF-126 cells exposed to DOX for 72 hours relative to the same cells in absence of DOX. The prevalence of this signature was evaluated across the intrinsic molecular subtypes of breast cancers using the previously published UNC breast cancer patient database (UNC337). P values were calculated by comparing gene expression means across all breast tumor subtypes. **B.** Kaplan–Meier survival estimates of relapse-free survival for the Merge 550 database (left panel), relapse-free survival and overall survival for the NKI 295 database (center and right panel, respectively). Patients were stratified into group I (red curves) and group II (blue curves) based on 2-way hierarchy clusters. P-values were obtained from the log-rank test. **C.** Mean expression analysis of the ATF-126 gene signature across breast cancer cell lines [21], showing the expression status of estrogen receptor (ER), Human Epidermal growth factor Receptor 2 (HER2) and progesterone receptor (PR).

We next examined this signature across intrinsic breast cancer subtypes, using the UNC337 tumor database comprising 337 breast tumor cases [20]. Our analysis shows that ATF-126 up-regulated targets that are found over-represented in the “Normal-like” intrinsic subtype of breast cancer (**Fig. 5A**). Both Normal-like and Luminal A are ER+ tumors associated with the best prognosis of all breast tumor subtypes [21]. In contrast, the Basal-like and Claudin-low carcinomas are mostly triple negative breast cancers (ER-PR-Her2-) associated with high resistance to chemotherapy and poor prognosis [20]. Claudin-low tumors have been recently discovered through large-scale microarray analysis of breast cancer specimens [20], [22]. The MDA-MB-231 cell line used in this study was originally described as Basal B and has been recently characterized as Claudin-low [20], [21]. Our finding that ATF-126-responsive genes are enriched in ER+ “Normal-like”, poorly aggressive tumors, correlates with the fact that ATF-126 induction in MDA-MB-231 confers a more benign, less tumorigenic phenotype. Consistent with this, we found that the ATF-126-up-regulated gene signature was indicative or a predictor of a better prognosis in breast cancer patients (**Fig. 5B**).

The relationship between the ATF-126-gene signature and ER status was further observed in available DNA-microarrays data of breast cancer cell lines [21]. **Fig. 5C** shows that the ATF-126-gene signature was found under-represented in Claudin-low and Basal-like ER- lines with the lowest enrichment found in the original MDA-MB-231 cell line. The same signature was over-represented in luminal ER+ cell lines, including the poorly aggressive lines MCF-7 and ZR75. Overall our microarray analysis provides support that ATF-126 initiates a transcriptional gene program resulting in a reprogramming of the original ER- aggressive Claudin-low MDA-MB-231 towards a less aggressive, and more “normal-like” breast cancer cell line. This is also consistent with the fact that high expression of *Maspin*, the primary target gene of ATF-126, is also associated with epithelial-like features [18].

**Figure 6:**
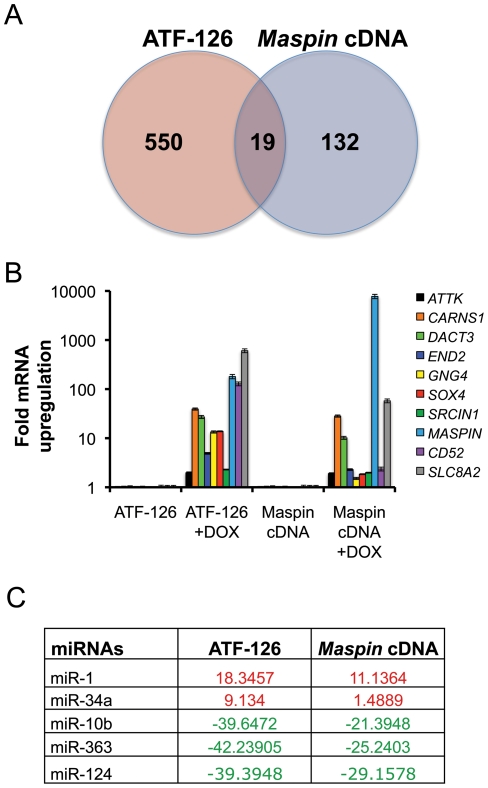
ATF-126 and *Maspin* cDNA co-regulated targets. **A.** Venn diagram indicating the intersecting up-regulated genes between the ATF-126 and the *Maspin* cDNA groups in DOX induced cells. **B.** Gene expression analyses of nine differentially up-regulated genes. The ATF-126 and *Maspin* cDNA stable cell lines were treated with either vehicle (−DOX) or DOX (+DOX) for a period of 72 hrs, and differences in expression quantified by qRT-PCR. Data was normalized to the −DOX cells, and represent an average of three independent experiments. **C.** Common MicroRNAs differentially regulated in the ATF-126 and the *Maspin* cDNA cell lines, as assessed by qRT-PCR. Data was normalized to the −DOX cells. Up-regulated miRNAs are indicated in red, and down-regulated miRNAs in green.

To dissect “bona-fide” downstream targets of *Maspin* in the 550-gene signature we cloned the *Maspin* cDNA into the same DOX-inducible retroviral vector, and gene expression microarrays were performed in the *Maspin* cDNA −DOX and +DOX transduced cell populations. 123 genes (**Table S3**) were found differentially up-regulated upon *Maspin* cDNA induction. Among these, 19 targets were shared with ATF-126 +DOX (**Fig. 6A** **,** **Table 1**). Nine of the most up-regulated *Maspin*-dependent candidate targets were further validated by qRT-PCR (**Fig. 6B**). These genes included potential therapeutic targets normally expressed in the neural system, such as negative regulators of oncogenic signaling (the epigenetic regulator of Wnt/β-catenin signaling (*DACT3*) and the SRC kinase signaling inhibitor 1 (*SRCIN1*)), putative tumor suppressors in cancer (*DACT3*, *SLC8A2*, *CARNS1*, *GNG4*, *END2*), and the apoptosis-associated tyrosine kinase *ATTK*. *Solute carrier family 8 (sodium/calcium exchanger), member 2* (*SLC8A2*), *Carnosine synthase* 1 (*CARNS1*), and *Dapper, antagonist of beta-catenin, homolog 3* (*DACT3*) were up-regulated by more than 10-fold in both, ATF-126 and *Maspin* cDNA cells, and thus, could represent novel “bona-fide” *Maspin*-dependent targets.

**Table 1:** Genes regulated with ATF-126 and Maspin cDNA.

GeneID	Name	Symbol
6543	solute carrier family 8 (sodium/calcium exchanger), member 2	SLC8A2
1043	CD52 molecule	CD52
6659	SRY (sex determining region Y)-box 4	SOX4
147906	dapper, antagonist of beta-catenin, homolog 3 (Xenopus laevis)	DACT3
1013	cadherin 15, type 1, M-cadherin (myotubule)	CDH15
162494	rhomboid, veinlet-like 3 (Drosophila)	RHBDL3
4093	SMAD family member 9	SMAD9
6615	snail homolog 1 (Drosophila)	SNAI1
285489	docking protein 7	DOK7
9148	neuralized homolog (Drosophila)	NEURL
3726	jun B proto-oncogene	JUNB
51207	dual specificity phosphatase 13	DUSP13
80725	SRC kinase signaling inhibitor 1	SRCIN1
57571	carnosine synthase 1	CARNS1
2786	guanine nucleotide binding protein (G protein), gamma 4	GNG4
29993	protein kinase C and casein kinase substrate in neurons 1	PACSIN1
9625	apoptosis-associated tyrosine kinase	AATK
126567	C2 calcium-dependent domain containing 4C	C2CD4C
1907	endothelin 2	EDN2

We next took advantage of ATF-126 and *Maspin* cDNA inducible cell lines to examine whether oncogenic and tumor suppressive microRNAs were differentially regulated upon DOX induction. We analyzed the expression of 90 miRNAs often dys-regulated in cancers, using a miRNA array platform by qRT-PCR. The expression of each miRNA was normalized to the MDA-MB-231 cell line. Interestingly, both, ATF-126 and *Maspin* cDNA, up-regulated miRNAs with potential tumor suppressive functions, such as miR-1 [23], [24] and miR-34 [25], while down-regulating oncogenes and metastasis promoters, including miR-10b [26] (**Fig. 6C**). In addition, we found that miR-124 which is required for somatic cells to reprogram to neural cells [27], and miR-363 (**Fig. 6C**), were down-regulated in both ATF-126 and *Maspin* cDNA. Thus, our target analysis suggests that ATF-126 activates downstream targets of *Maspin* resulting in an up-regulation of potential tumor suppressors, and down-regulation of oncogenic and pro-metastatic pathways.

**Figure 7:**
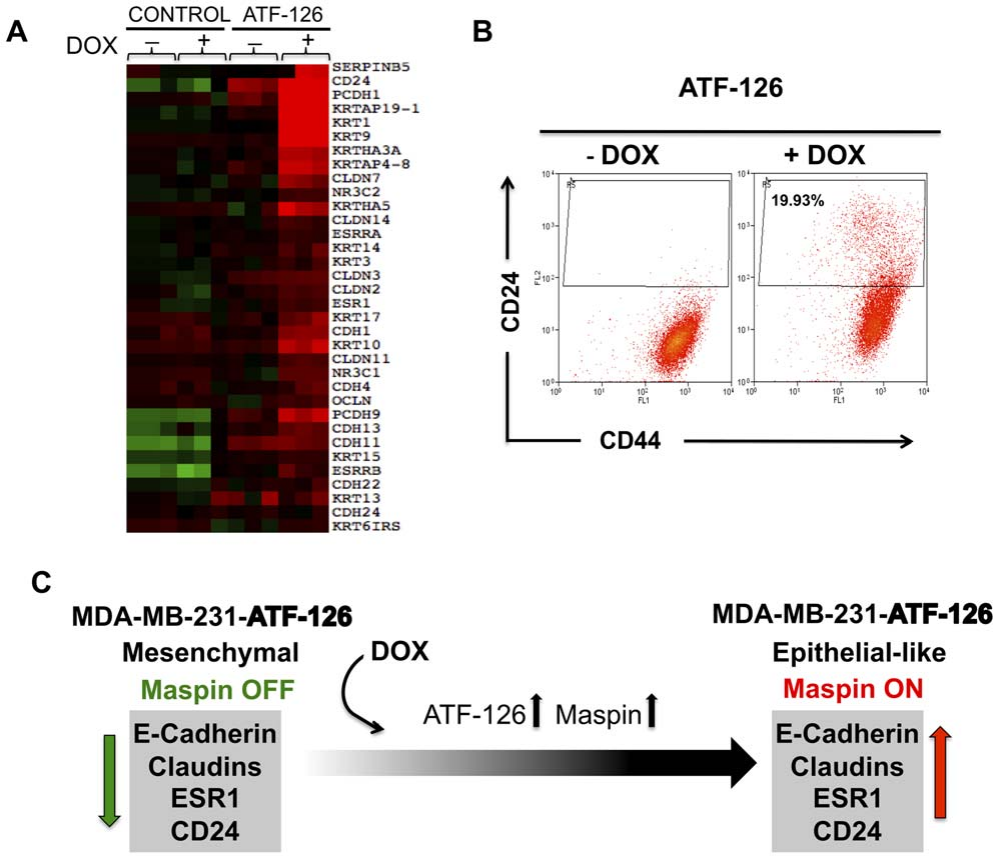
ATF-126 regulates markers associated with decreased tumorigenicity and metastasis. **A.** Microarray expression analysis of selected epithelial markers in CONTROL and ATF-126 cells, in presence or absence of DOX. Cells were collected 72 hours after DOX induction. Arrays were performed in triplicate with three different biological replicates using Agilent 44 k arrays. The Array tree was derived from an unsupervised hierarchical clustering and the gene list is shown in **Table S1**. Each colored square on the upper right represents the relative mean transcript abundance (in log2 space) with highest expression being red, average expression being black, and lowest expression being green. **B.** Induction of ATF-126 by DOX modifies the tumor initiating cell signature CD44^+^CD24^−^. Representative flow cytometric analysis of CD44 and CD24 expression levels in ATF-126 −DOX and +DOX cells collected 72 hours after treatment. The forward scatter (FCS) channel was plotted in y-axis and the fluorescence of the cell surface antigens in the x-axis. The gate in the +DOX panel illustrates the generation of a novel CD24+ population upon induction of ATF-126. **C.** A model illustrating a potential mechanism by which ATF-126 could reprogram a mesenchymal, Claudin-low MDA-MB-231 cell line towards a more epithelial-like phenotype.

Claudin-low carcinomas and representative cell lines are characterized by a down-regulation of epithelial junction proteins, such as cadherins and claudins [20]. Interestingly, multiple panels of epithelial markers, such as E-Cadherin (*CDH1*), Claudin 3 and 7, Ocludins, and keratins, were re-activated upon ATF-126 expression (**Fig. 7A**). In addition, ATF-126 led to the generation of a CD24 positive population (**Fig. 7B**). CD24 is expressed in many ER+ tumor cell lines, while its expression is absent in some basal and Claudin-low cell lines (**Fig. S2**). CD44+/CD24- is considered a cancer stem cell or tumor initiating cell signature, and high CD44/CD24 ratios are characteristic of aggressive Claudin-low tumors and cell lines [20]. The reactivation of CD24 by ATF-126 suggests that ATF-126 could decrease the tumorigenic potential of MDA-MB-231 cells. In summary, the above results suggested that ATF-126 was able to initiate a transcriptional program resulting in a reprogramming of a more mesenchymal, Claudin-low phenotype, towards a more normal-like, epithelial-like, and less aggressive breast tumor line (**Fig. 7C**).

## Discussion

In this paper we have investigated the ability of the ATF-126, designed to up-regulate *Maspin*, to decrease tumor growth and colonization of an aggressive MDA-MB-231 line. We found that induction of ATF expression *in vivo* resulted in 50% reduction in tumor growth. In addition, ATF expression abolished the capability of the breast cancer to colonize the lungs.

The approach used in this work facilitated the tight control of the ATF expression both in cell culture and in a breast cancer xenograft model. As a direct target of the ATF, we found that the *Maspin* transcript was induced with very similar time course kinetics than the ATF. Interestingly, removal of DOX from the cell culture media resulted in a quick decay of ATF-126 expression. However, we found that *Maspin* expression was still retained for at least four cell generations even in complete absence of ATF-126 mRNA expression. In the same time window of *Maspin* transcriptional activation, the ATF-126 +DOX cells maintained their proliferation defects, suggesting that the growth inhibition phenotype could be propagated for several cell generations upon DOX removal. The slow decay of *Maspin* expression and the maintenance of growth inhibition in absence of ATF expression could be the result of the modification of the epigenetic status of the *Maspin* promoter upon binding of the ATF. We have reported that two ATFs, ATF-126 and ATF-97, reduced DNA methylation levels in the *Maspin* promoter [13]. In addition, this demethylation effect was directional and depended upon de orientation of VP64 along the promoter. In this regard, it is possible that the slow *Maspin* decay in absence of ATF expression could reflect a time delay by which the endogenous epigenetic and/or transcriptional mechanisms restore *Maspin* silencing. To address this possibility, we are presently analyzing methylation patterns upon removal of DOX at different time points. Future engineering of ATFs should maximize this demethylation effect, thereby increasing the potency and therapeutic window of ATFs targeting tumor suppressor gene promoters. This unique engineering aspect of ATFs could facilitate the long-term, hereditary, and stable transmission of tumor suppression (“phenotypic memory”) over cell generations by targeted remodeling of silenced chromatin.

Our results *in vivo* demonstrate that induction of ATF-126 in animal models led to a 50% reduction in breast cancer cell growth. Long-term removal of DOX from the diet of the animals resulted in a re-establishment of the tumors *in vivo*. As it was observed in our analysis *in vitro*, it is possible that long-term absence of ATF expression results in re-establishment of *Maspin* silencing. This is in contrast with our colonization model of breast cancer, in which removal of DOX from the ATF-126 induced animals did not result in a recurrence of metastasis. These results could be explained because most circulating tumor cells or cells that underwent an early colonization in the lungs were effectively targeted by the ATF. Nevertheless, we do not know at present if the ATF-126 will be also effective in suppressing well-established metastases and this will require further investigation.

In order to investigate potential downstream genetic signatures mediating the inhibition of tumor growth and colonization, we performed DNA arrays. Our genome-wide analysis revealed that ATF-126 up-regulated a 550-gene signature that was found over-represented in the Normal-like intrinsic subtype of breast cancer, and as well as in luminal A breast cancer cell lines. Together with Luminal A, Normal-like breast cancers are associated with ER expression. These tumors are small, mostly found in post-menopausal women, tend to have normal p53 status, and are known to be genetically more stable than other tumor subtypes [17], [18]. Hence, Normal-like and Luminal A tumors both have a significant higher survival upon endocrine adjuvant therapy treatments after surgery as compared to other subtypes of breast cancers. As expected from its over-representation in Normal-like ER+ tumors, we found that the 550-gene signature predicted favorable prognosis for breast cancer patients.

In our cell line analysis, the 550-gene signature was under-represented in the original MDA-MB-231 line as well as in ER- breast cancer lines, while it was highly over-represented in ER+ luminal lines. Although Normal-like cancer cell lines were absent in the cell line database of Neve *et al.* [21] it is highly possible that this signature will also be over-expressed in normal breast and Normal-like tumor cell lines. Consistent with this idea, we found that many junction proteins highly expressed in normal breast epithelial preparations, such as claudins, cadherins and ocludins, were induced upon DOX treatment. This reactivation of junction proteins suggests that ATF-126 was able to reprogram a highly invasive, Claudin-low, and mesenchymal breast cancer line towards a more Normal-like or epithelial-like, and less invasive cancer cell line. Furthermore, flow cytometric analysis of ATF-126 induced cells revealed a new CD24 positive population. Since the CD44^+^/CD24^−^ signature has been associated with tumor initiation, the up-regulation of CD24 population suggest that ATF-126 could decrease the tumor initiating ability of the original line MDA-MB-231.

The above phenotypic consequences of ATF-126 reactivation, inhibition of tumor growth and metastatic potential as well as gain of epithelial features, are all consistent with the documented functions of the target gene *Maspin*. The fact that both the *Maspin* shRNA and the *Maspin* siRNA were able to rescue the cell growth phenotype in ATF-126 transduced cells demonstrated that the phenotype of ATF-transduced cells was dependent on *Maspin*, and not on potential off target effects.

The molecular targets by which *Maspin* exerts its tumor/metastasis suppressive functions are still under investigation. *Maspin* cDNA over-expression results in re-activation of multiple pathways involved in tumor suppression and apoptosis, motility, and cell adhesion [18],[16]. In agreement with these functions of *Maspin*, we have found that tight junction, cell adhesion, and multiple tumor suppressive pathways were activated by ATF-126.

To dissect the downstream targets of ATF-126 that were “bona fide” downstream targets of *Maspin*, the *Maspin* cDNA was cloned into the same inducible retroviral vector, and genome-wide transcriptional profiles were compared between the un-induced and induced cells. An overlapping 19-target gene signature between the ATF-126 and the *Maspin* cDNA expressing cells was built, which represents “high confidence” hits by which *Maspin* could exert its mechanism of function in Claudin-low breast cancer cells. It is important to note that ATF-126 and *Maspin* cDNA represent two different mechanisms to up-regulate target gene expression; whereas the *Maspin* cDNA over-expresses the exogenous transgene form of *Maspin*, the ATF reactivates the endogenous gene, which results in up-regulation the physiologically relevant isoform, at “normal” cellular levels. In addition, unlike cDNA over-expression, which is predominantly cytoplasmic, the ATF-126 also results in substantial activation of the nuclear form of *Maspin* (*manuscript in preparation*), which mediates tumor and metastasis suppressive functions [28]. Thus, it is not surprising that transcriptional profiles of ATF-126 and *Maspin* cDNA were overlapping, yet not identical. Definitive analysis of genome-wide binding specificity of ATF-126 will require the integration of ChIP-seq data, which is underway. Overall, our results outlined the importance of using several methodologies to up-regulate *Maspin*, which allowed the dissection of potential therapeutic targets downstream the *Maspin* cascade.

Three targets belonging to this overlapping 19-gene signature were up-regulated by 10–100 fold over un-induced cells: *Solute carrier family 8 (sodium/calcium exchanger), member 2* (*SLC8A2*), *Carnosine synthase* 1 (*CARNS1*), and *Dapper, antagonist of beta-catenin, homolog 3* (*DACT3*). These genes represent novel *Maspin* targets with potential tumor suppressor activity. *SLC8A2* encodes a Na(+)/Ca(2+) exchanger, participating in intracellular Ca(2+) homeostasis, and its expression is restricted to the brain. Interestingly, *SLC8A2* is found silenced by methylation in human gliomas where it has been proposed to act a tumor suppressor gene [29]. *CARNS1* belongs to the ATP-grasp family of ATPases, and catalyzes the formation of carnosine (beta-alanyl-L-histidine) and homocarnosine (gamma-aminobutyryl-L-histidine). Carnosine is a naturally occurring substance discovered more than a hundred years ago, and is present in the mammalian brain and skeletal muscle [30]. Although is function is still under investigation, Carnosine has been proposed to have a protective effect against oxidative stress and represents a potential therapeutic agent for treatment of aging and Alzheimer's disease [31]. More recently Carnosine has been shown to inhibit proliferation of human brain cancer cells *in vitro* [32]. In 2010 a report showed that Carnosine retarded tumor growth *in vivo* in a NIH3T3-HER2/neu mouse model [33]. Thus, our results suggest that *CARNS1* could potentially represent a *Maspin*-dependent tumor suppressor enzyme in Claudin-low carcinomas. *DACT3* is expressed in the embryonic CNS, and is a negative regulator of Wnt/β-catenin signaling [34]. *DACT3* is down-regulated in colorectal cancers by epigenetic mechanisms including histone methylation and deacetylation [35]. Given the importance of Wnt/β-catenin in the mammary gland tumorigenesis in triple negative, mesenchymal breast cancers [36], we speculate that *DACT3* represents a potential therapeutic target for these carcinomas.

ATF-126 and in less extent *Maspin* cDNA +DOX cells up-regulated the *protein gamma-4 subunit/guanine nucleotide-binding protein 4* (*GNG4*) gene, a brain-specific protein [37] with potential tumor suppressor activity, which, like *Maspin* [38], is up-regulated by hypoxia factors [39]. Another unexplored potential target of *Maspin* in the 19-gene signature is the *Apoptosis-Associated Tyrosine kinase* (*AATYK*), a protein kinase predominantly expressed in the nervous system. *AATYK* regulates apoptosis, neurite growth, and differentiation of cerebellar granule cultures [40]. Lastly, ATF-126, and in less extent *Maspin* cDNA, up-regulated the *SRC kinase signaling inhibitor 1* (*SRCIN1*), a gene encoding a novel Src-binding protein that regulates Src activation. Gain and loss of function approaches in breast and colon cancer cells demonstrated that *SRCIN1* inhibits EGFR and Erk1/2 signaling, blocking scatter and proliferation of cancer cells [41]. In neurons, *SRCIN1* is predominantly localized to dendritic spines and enriched in the postsynaptic density, where it modulates spine shape *via* regulation of the actin cytoskeleton [42].

In addition to potential tumor/metastasis suppressor ORFs naturally expressed in neural cells, both ATF-126 and *Maspin* cDNA up-regulated miRNAs previously associated with tumor suppression in many types of cancers, including miR-1 [23], [24] and miR-34 [25]. Intriguingly again, miR-1 is naturally expressed in dorsal root ganglion neurons where it has a role in modulating neurite outgrowth [43]. Similarly, miR-34 has been shown to target Actin in mouse neuronal cells [44]. In addition to activation of potential tumor suppressive miRNAs, both ATF-126 and *Maspin* cDNA down-regulated putative oncogenes, including miRNA-10b.

The up-regulation of epigenetically silenced genes and miRNAs normally expressed in the nervous system is intriguing. Once over-expressed in tumors, these targets could be involved in differentiation of tumor cells, and metastasis inhibition. As a member of the *SERPIN* gene superfamily, nuclear *Maspin* is physically associated with Histone Deacetylase 1 (HDAC1) and functions as an HDAC inhibitor, remodeling chromatin and mediating gene reactivation [28], [45]. This is in agreement with our data, and could mechanistically explain the epigenetic reactivation of multiple tumor suppressors epigenetically silenced in poorly differentiated tumor cells, and the overall reprogramming of a mesenchymal line towards a more benign, more differentiated, and less aggressive cell line. Subsequent analysis in our identified *Maspin* targets will reveal whether *Maspin* directly binds the promoter of these genes, resulting in tumor and metastasis suppression. We are presently addressing the potential of ATFs to be delivered in breast tumors and metastasis, using nanoparticle-based strategies (*manuscript in preparation*). Another line of investigation will ascertain if ATFs can increase the sensitivity of ER- tumors to anti-cancer drugs used in the clinic, such as anti-estrogens.

## Materials and Methods

### Cell lines

The human breast cancer cell line MDA-MB-231 (ATCC, Cat. No. HTB-26) and all stable cell lines derived from it were cultured in DMEM medium supplemented with 10% fetal bovine serum and grown at 37°C in a humidified 5% CO_2_ incubator.

### Development of a double stable Tet-On cell lines

The ZF-126, the ATF-126, and the *Maspin* cDNA were cloned into the p-RetroX-Tight (Cat. Num. 632104, CloneTech, Mountain View, CA, US), and delivered into the MDA-MB-231-LUC cells by retroviral transduction. After transduction the cells were placed under selection with puromycin (1 µg/ml) and geneticin (800 µg/ml) for ten days. The CONTROL (p-RetoX-Tight empty vector), ZF-126 (p-RetoX-Tight-ZF-126), the ATF-126 (p-RetoX-Tight-ATF-126), and the *Maspin* cDNA (p-RetoX-Tight-*Maspin*) stable cell lines were expanded and used for *in vitro* and *in vivo* experiments.

### ShRNA stable cell lines

Scramble and *Maspin* shRNAs (Open Biosystems) were co-transfected with pMDG.1 into 293TGag-Pol cells to produce retroviral particles. Transfection was done using Lipofectamine system. The viral supernatant was used to infect the MDA-MB-231-ATF-126 stable cell line. Cells were treated with hygromycin B (100 µg/ml) for 10 days.

### siRNA *Maspin* gene knock-down

The p-RetoX-Tight-ATF-126 stable cell line was reverse-transfected with either 50 nM of *Maspin* siRNA smart pool (4 siRNAs/pool) or mismatch siRNA, and complexed with dharmaFECT reagent (all siRNAs and reagents from Dharmacon, Chicago, IL, USA). Transfection conditions were optimized using a cytotoxic siRNA targeted against human ubiquitin B (Dharmacon). Cells were induced with DOX for 24 hours, then 2.5×10^5^ cells/well were transfected in 6-well plates, and DOX was added. Cells were maintained in transfection media for 72 hours, and subjected qRT-PCR analysis.

Gene expression, western blot, proliferation, Immunofluorescence and apoptosis assays were performed as described [13]. Primers and probes for gene expression assays are shown in **Table S4**. Primary and secondary antibodies from the corresponding companies were applied at the concentrations shown in **Table S5**.

### Quantification of cell death

Cells were collected and centrifuged 5 min at 100×g, then resuspended in 0.2% trypan blue. Cells were incubated 3 min at room temperature and counted on a hemacytometer. The percentage of cell death was calculated by comparing all samples counts with the ATF-126 −DOX cells.

### Subcutaneous Injections

Female SCID mice (age 4 weeks) were purchased from Taconic Farms and housed under pathogen-free conditions. The Institutional Animal Care and Used Committee (IACUC) at the University of North Carolina at Chapel Hill approved all experiments described herein. MDA-MB-231-Control-LUC or MDA-MB-231-ATF126-LUC cells (1×10^6^) were collected and re-suspended with matrigel (BD Bioscience, San Diego, CA, US) 1∶1 volume ratio in a total volume of 100 µl. The cell-matrigel mixture was injected into the mouse flank. Tumor growth was monitored by caliper twice a week. When the tumor reached a size of approximately 0.5 cm, doxycycline (+DOX) was administered to mice in form of green food pellet (200 mg/Kg of mice chow) for a period of 25 days. For the DOX removal group, the DOX food was replaced by normal food. During the entire experiment the mice weight was monitored to verify whether toxicity occurred. After DOX treatment, the tumor volume was monitored both by caliper and Bioluminescence imaging (BLI) as described [13]. Assessment of tumor shrinkage was monitored one day previous to DOX induction and the day of tumor collection.

### Tail-vein

Three days before the injections mice were kept in normal or DOX+ diet. Animals were injected via tail-vein with either 1×10^5^ MDA-MB-231-Control-LUC or MDA-MB-231-ATF126-LUC cells in 100 µl of PBS. BLI imaging was performed as described [13]. Assessment of tumor growth was monitored *in vivo* once a week for up to 8 weeks.

### Flow cytometry

Cells were stained with primary antibodies anti-CD24-conjugated with phycoerythrin (PE) and anti-CD44 conjugated with Fluorescein isothiocyanate (FITC) (BD Bioscience, San Diego, CA, US) following the manufacturer's recommendations. Analysis was performed using a FACScalibur flow cytometer and the CellQuestTM software.

### Gene expression microarrays

A total of six cell lines were used for gene expression analyses: CONTROL −DOX, CONTROL +DOX, ATF-126 −DOX, ATF-126 +DOX (all with 3 technical replicates), p-RetoX-Tight-Maspin −DOX, and p-RetoX-Tight-Maspin +DOX (with 2 technical replicates). For each cell line, total RNA was purified, amplified, labeled, and hybridized [46] using Agilent Agilent 4×44 K oligo microarrays (Agilent Technologies, United States). All microarray data is available in the Gene Expression Omnibus (GEO) Database (http://www.ncbi.nlm.nih.gov/geo/query/acc.cgi?token=nnolvkqsosuwwlm&acc=GSE27842). The probes/genes were filtered by requiring the lowest normalized intensity values in both −DOX and +DOX samples to be >10. The normalized log2 ratios (Cy5 sample/Cy3 control) of probes mapping to the same gene were averaged to generate independent expression estimates. We also used available microarrays from the breast cancer cell lines [21], the UNC337-patient [20], the MERGE 550-patient dataset [47] and the NKI (295 patients [48], [49]). All microarray cluster analyses were displayed using Java Treeview version 1.1.3. Average-linkage hierarchical clustering was performed using Cluster v2.12 [50]. ANOVA tests for gene expression data were performed using R (http://cran.r-project.org).

### ATF-126/Maspin cDNA gene signatures

In order to build an ATF-126 signature, we selected those genes that were significantly differentially expressed between ATF-126+DOX and ATF-126 −DOX using SAM, with <1% FDR. The resulting up-regulated gene list is shown in **Table S1**.

In order to determine the genes up-regulated with the *Maspin* cDNA, two biological replicates of the non-induced cells (−DOX) and cells induced with DOX were subjected to DNA microarray analyses, as described above. Gene expression values from the *Maspin* cDNA −DOX (N = 2) was subtracted from the *Maspin* cDNA +DOX (N = 2), and the 132 up-regulated genes are shown in **Table S5**.

### MicroRNA microarrays

Total RNA was purified from the ATF-126 and the *Maspin* cDNA cells treated with either vehicle (−DOX) or DOX for 72 hours using Trizol. Small RNAs were enriched using the miRNeasy Mini Kit (SA Biosciences, Frederick, MD, USA), and cDNA was generated with the RT2 miRNA First Strand Kit (SABiosciences, Frederick, MD, USA). Samples were subjected to MAH-102-C Micro-RNA arrays (SABiosciences, Frederick, MD, USA), and data was normalized to the vehicle treated cells (−DOX). Expression of miR-10b, miR-1, miR-34a, miR363, and miR-124 was validated in two independent assays using hydrolysis probes (**Table S4**).

### Prediction of relapse-free survival (RFS)

To determine if the 550-gene signature was represented in cancer patients from whom clinical data was available, we used two patient data sets: the NKI-295 and the MERGE-550. First, we examined the expression of the 550 genes in the MERGE-550 database and found that 322 out of the 550 genes were detected. A 2-way hierarchy cluster stratified the 322 patients into 2 groups (cluster I and cluster II; **Fig. S3**). Patients in cluster II had a significant better relapse free survival outcome (in 7 years follow-up) than patients in cluster I. Second, we repeated the analysis using the NKI dataset and found 444 genes. A 2-way hierarchy cluster stratified the 295 patients into 2 groups (cluster I and cluster II; **Fig. S4**). Here again, patients in cluster II had a significant improved relapse free survival outcome and overall survival outcome (in 18 years follow-up) relative to patients in cluster I.

## Supporting Information

Figure S1 **Generation of an inducible ATF expression system to monitor breast tumor and metastasis.** **A.** Schematic representation of ATF-126 comprising the six Zinc Finger (ZF) specific DNA-binding domains and the VP64 transactivator domain. ATF-126 was targeted against a unique 18-base pair site in the *Maspin* promoter. **B.** ATF-126 induction to monitor breast tumor progression. ATF-126 was cloned into pRetroX-Tight inducible vector system. The pRetroX-Tight vector is composed of a modified tetracycline response element (TRE_Mod_) and a minimal CMV promoter (CMV_min_). The activator protein is a tetracycline–controled transactivator (rtTA), which binds to the TRE_Mod_ sequences in presence of Doxocycline (DOX). Viral particles were prepared and MDA-MB-231-LUC cells (engineered with a luciferase reporter) were transduced to generate stable cell lines. ATF-126 was induced both *in vitro* and *in vivo* with the chemical inducer DOX.
(TIFF)

Figure S2 **Expression levels of CD44 and CD24 in a panel of breast cancer cell lines, as assessed by flow cytometry.**
(TIFF)

Figure S3 **Gene clustering showing the 322 genes from the ATF-126-550 gene signature present in the MERGED-550 patient dataset.** Two-way hierarchy cluster stratified the 550 patients into two groups (cluster I and cluster II); patients in cluster II had a significant better relapse free survival outcome (in 7 years follow-up) than patients in cluster I. Each colored square on the upper right represents the relative mean transcript abundance (in log2 space) with highest expression being red, average expression being black, and lowest expression being green.
(TIFF)

Figure S4 **Gene clustering showing the 444 genes from the ATF-126-550 gene signature present in the NKI-295 patient dataset.** Two-way hierarchy cluster stratified the 295 patients into 2 groups (cluster I and cluster II); patients in cluster II had a significant better relapse free survival outcome and overall survival outcome (in 18 years follow-up) than patients in cluster I. Each colored square on the upper right represents the relative mean transcript abundance (in log2 space) with highest expression being red, average expression being black, and lowest expression being green.
(TIFF)

Table S1 **Genes up-regulated in ATF-126 +DOX versus −DOX.**
(DOC)

Table S2 **Pathway analysis of the 550-gene signature using the David database (** http://david.abcc.ncifcrf.gov/ **).**
(DOC)

Table S3 **Antibodies used in this study.**
(DOCX)

Table S4 **Primers and probes used in this study.**
(DOCX)

Table S5 **Genes differentially regulated with the** ***Maspin*** **cDNA.**
(DOC)
