# Supplementary figures and images for: Suppression of Breast Tumor Growth and Metastasis by an Engineered Transcription Factor
Source: PLoS One. 2011 Sep 13;6(9):e24595. doi: 10.1371/journal.pone.0024595 (PMC3172243; doi:10.1371/journal.pone.0024595)

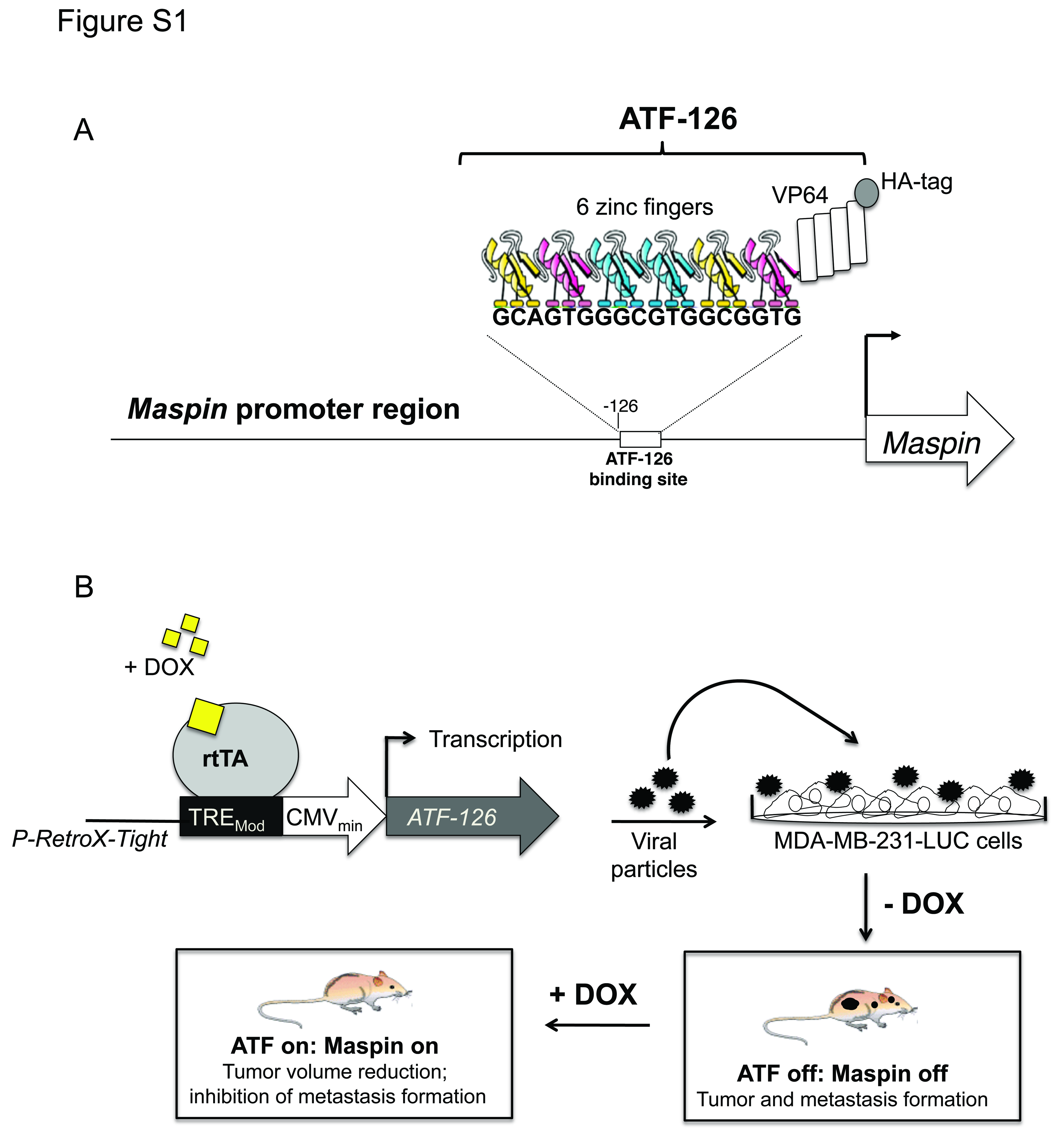

Supplement: Figure S1 — Generation of an inducible ATF expression system to monitor breast tumor and metastasis. A. Schematic representation of ATF-126 comprising the six Zinc Finger (ZF) specific DNA-binding domains and the VP64 transactivator domain. ATF-126 was targeted against a unique 18-base pair site in the Maspin promoter. B. ATF-126 induction to monitor breast tumor progression. ATF-126 was cloned into pRetroX-Tight inducible vector system. The pRetroX-Tight vector is composed of a modified tetracycline response element (TREMod) and a minimal CMV promoter (CMVmin). The activator protein is a tetracycline–controled transactivator (rtTA), which binds to the TREMod sequences in presence of Doxocycline (DOX). Viral particles were prepared and MDA-MB-231-LUC cells (engineered with a luciferase reporter) were transduced to generate stable cell lines. ATF-126 was induced both in vitro and in vivo with the chemical inducer DOX. (TIFF) [file pone.0024595.s001.tif]

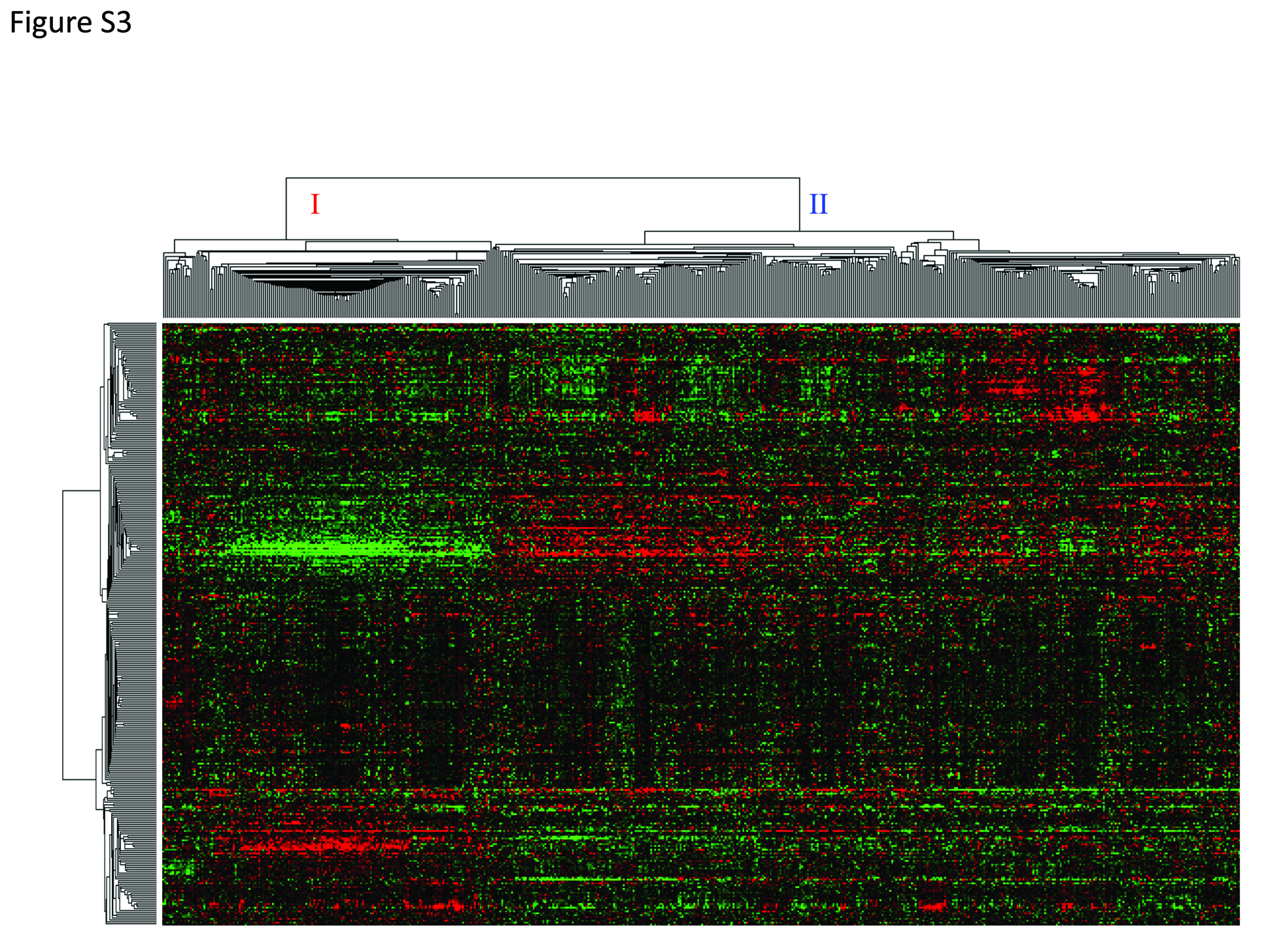

Supplement: Figure S3 — Gene clustering showing the 322 genes from the ATF-126-550 gene signature present in the MERGED-550 patient dataset. Two-way hierarchy cluster stratified the 550 patients into two groups (cluster I and cluster II); patients in cluster II had a significant better relapse free survival outcome (in 7 years follow-up) than patients in cluster I. Each colored square on the upper right represents the relative mean transcript abundance (in log2 space) with highest expression being red, average expression being black, and lowest expression being green. (TIFF) [file pone.0024595.s003.tif]

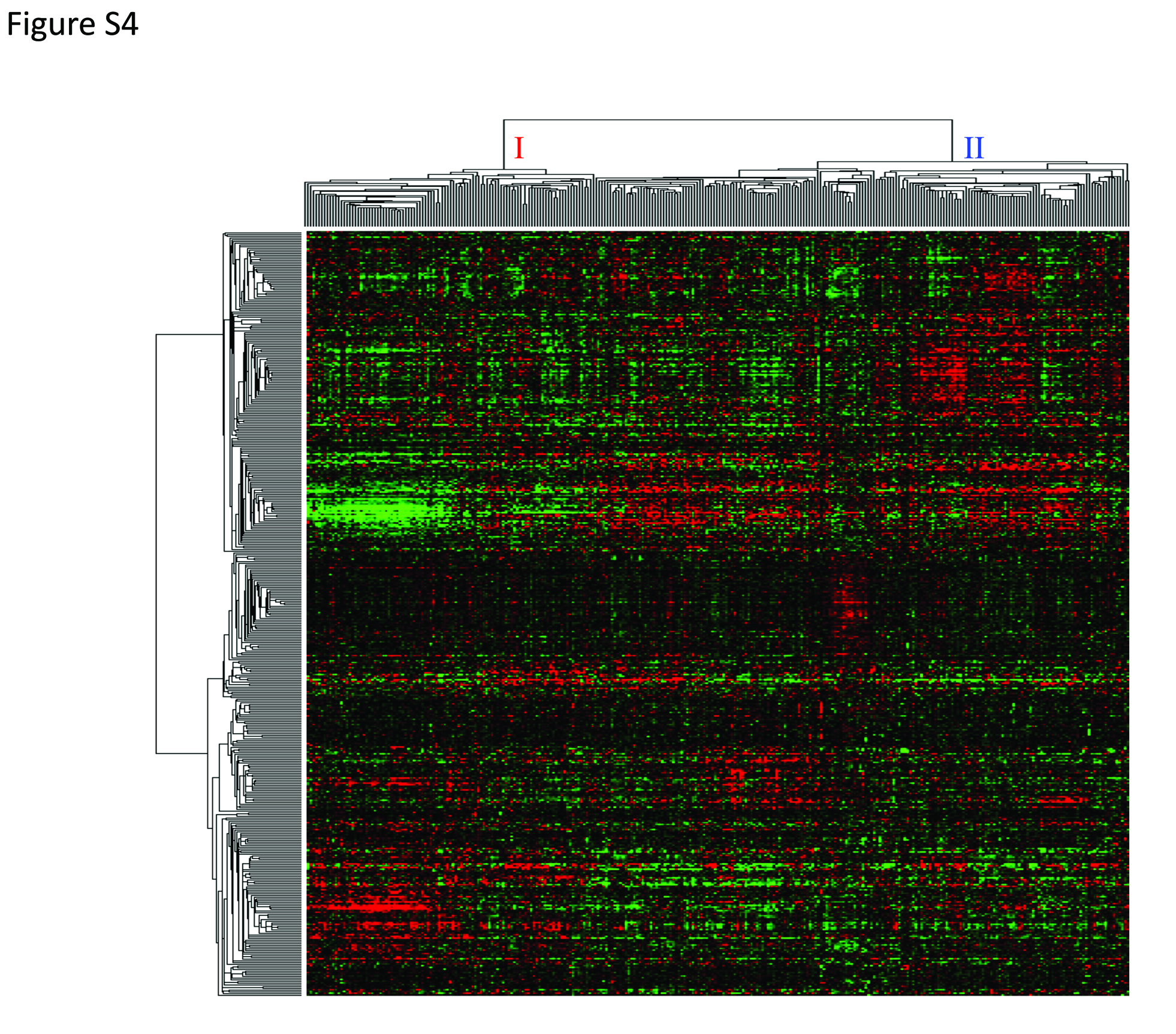

Supplement: Figure S4 — Gene clustering showing the 444 genes from the ATF-126-550 gene signature present in the NKI-295 patient dataset. Two-way hierarchy cluster stratified the 295 patients into 2 groups (cluster I and cluster II); patients in cluster II had a significant better relapse free survival outcome and overall survival outcome (in 18 years follow-up) than patients in cluster I. Each colored square on the upper right represents the relative mean transcript abundance (in log2 space) with highest expression being red, average expression being black, and lowest expression being green. (TIFF) [file pone.0024595.s004.tif]
