# Supplementary material for: Suppression of Breast Tumor Growth and Metastasis by an Engineered Transcription Factor
Source: PLoS One. 2011 Sep 13;6(9):e24595. doi: 10.1371/journal.pone.0024595 (PMC3172243; doi:10.1371/journal.pone.0024595)
Supplement: Table S1 — Genes up-regulated in ATF-126 +DOX versus −DOX. (DOC) [file pone.0024595.s005.doc]

**Table S1. Genes up-regulated in ATF-126 +DOX versus -DOX**

| **Gene ID** | **Symbol** | **Cytoband** | **Fold Change** | **Name** |
| --- | --- | --- | --- | --- |
| 10742 | RAI2 | Xp22 | 262.4597708 | retinoic acid induced 2 |
| 245972 | ATP6V0D2 |  | 124.571768 | ATPase, H+ transporting, lysosomal 38kDa, V0 subunit d2 |
| 6690 | SPINK1 | 5q32 | 83.4206896 | serine peptidase inhibitor, Kazal type 1 |
| 26053 | AUTS2 | 7q11.22 | 145.3571606 | autism susceptibility candidate 2 |
| 4319 | MMP10 | 11q22.3 | 65.31175785 | matrix metallopeptidase 10 (stromelysin 2) |
| 624 | BDKRB2 | 14q32.1-q32.2 | 45.18021288 | bradykinin receptor B2 |
| 83481 | EPPK1 | 8q24.3 | 47.58164793 | epiplakin 1 |
| 387640 | C10orf140 | 10p12.31 | 46.13106412 | chromosome 10 open reading frame 140 |
| 219699 | UNC5B | 10q22.1 | 36.91153936 | unc-5 homolog B (C. elegans) |
| 3745 | KCNB1 | 20q13.2 | 34.06659452 | potassium voltage-gated channel, Shab-related subfamily, member 1 |
| 152789 | JAKMIP1 | 4p16.1 | 33.98909397 | janus kinase and microtubule interacting protein 1 |
| 123591 | C15orf27 | 15q24.2 | 30.46329307 | chromosome 15 open reading frame 27 |
| 4322 | MMP13 | 11q22.3 | 51.4534638 | matrix metallopeptidase 13 (collagenase 3) |
| 6543 | SLC8A2 | 19q13.3 | 45.61174099 | solute carrier family 8 (sodium/calcium exchanger), member 2 |
| 79570 | NKAIN1 | 1p35.2 | 28.7468771 | Na+/K+ transporting ATPase interacting 1 |
| 3398 | ID2 | 2p25 | 43.15133634 | inhibitor of DNA binding 2, dominant negative helix-loop-helix protein |
| 9355 | LHX2 | 9q33.3 | 36.70477056 | LIM homeobox 2 |
| 93953 | ACRC | Xq13.1 | 46.48414023 | acidic repeat containing |
| 10826 | C5orf4 | 5q31-q32 | 26.11246352 | chromosome 5 open reading frame 4 |
| 1043 | CD52 | 1p36 | 25.79117427 | CD52 molecule |
| 170850 | KCNG3 | 2p21 | 26.47697993 | potassium voltage-gated channel, subfamily G, member 3 |
| 84249 | PSD2 | 5q31.2 | 21.24354304 | pleckstrin and Sec7 domain containing 2 |
| 1880 | GPR183 | 13q32.3 | 27.71673059 | G protein-coupled receptor 183 |
| 84206 | MEX3B | 15q25.2 | 31.25106245 | mex-3 homolog B (C. elegans) |
| 337882 | KRTAP19-1 | 21q22.1 | 23.52662097 | keratin associated protein 19-1 |
| 23507 | LRRC8B | 1p22.2 | 19.59843659 | leucine rich repeat containing 8 family, member B |
| 80853 | JHDM1D | 7q34 | 22.29262257 | jumonji C domain containing histone demethylase 1 homolog D (S. cerevisiae) |
| 2049 | EPHB3 | 3q21-qter | 24.63549691 | EPH receptor B3 |
| 140862 | ISM1 | 20p12.1 | 25.46305001 | isthmin 1 homolog (zebrafish) |
| 23769 | FLRT1 | 11q12-q13 | 30.73193424 | fibronectin leucine rich transmembrane protein 1 |
| 4094 | MAF | 16q22-q23 | 21.92492496 | v-maf musculoaponeurotic fibrosarcoma oncogene homolog (avian) |
| 11037 | STON1 | 2p16.3 | 20.0828051 | stonin 1 |
| 356 | FASLG | 1q23 | 22.05901239 | Fas ligand (TNF superfamily, member 6) |
| 441801 | LOC441801 | 17p13.3 | 17.39780426 | LOC441801 |
| 253738 | EBF3 | 10q26.3 | 18.04535419 | early B-cell factor 3 |
| 4674 | NAP1L2 | Xq13 | 23.72495315 | nucleosome assembly protein 1-like 2 |
| 1649 | DDIT3 | 12q13.1-q13.2 | 18.96586587 | DNA-damage-inducible transcript 3 |
| 140706 | C20orf160 | 20q11.2 | 17.15573657 | chromosome 20 open reading frame 160 |
| 9244 | CRLF1 | 19p12 | 18.10103141 | cytokine receptor-like factor 1 |
| 9518 | GDF15 | 19p13.11 | 19.13753795 | growth differentiation factor 15 |
| 6657 | SOX2 | 3q26.3-q27 | 17.91173685 | SRY (sex determining region Y)-box 2 |
| 6286 | S100P | 4p16 | 23.38751489 | S100 calcium binding protein P |
| 143686 | SESN3 | 11q21 | 17.22581097 | sestrin 3 |
| 440934 | LOC440934 | 2q36.1 | 20.11221399 | hypothetical LOC440934 |
| 80726 | KIAA1683 | 19p13.1 | 17.17613265 | KIAA1683 |
| 22843 | PPM1E | 17q22 | 16.98473338 | protein phosphatase, Mg2+/Mn2+ dependent, 1E |
| 2977 | GUCY1A2 | 11q21-q22 | 17.97956025 | guanylate cyclase 1, soluble, alpha 2 |
| 5588 | PRKCQ | 10p15 | 17.2634845 | protein kinase C, theta |
| 54463 | FAM134B | 5p15.1 | 16.52601144 | family with sequence similarity 134, member B |
| 23462 | HEY1 | 8q21 | 19.18180617 | hairy/enhancer-of-split related with YRPW motif 1 |
| 3857 | KRT9 | 17q21.1-q21.2 | 16.72962652 | keratin 9 |
| 4703 | NEB | 2q22 | 18.57126263 | nebulin |
| 58495 | OVOL2 | 20pter-q11.23 | 17.24771493 | ovo-like 2 (Drosophila) |
| 5567 | PRKACB | 1p36.1 | 15.78884063 | protein kinase, cAMP-dependent, catalytic, beta |
| 222537 | HS3ST5 | 6q21 | 19.02325996 | heparan sulfate (glucosamine) 3-O-sulfotransferase 5 |
| 199675 | C19orf59 | 19p13.2 | 14.36009169 | chromosome 19 open reading frame 59 |
| 5017 | OVOL1 | 11q13 | 31.82567402 | ovo-like 1(Drosophila) |
| 51450 | PRRX2 | 9q34.1 | 16.43408426 | paired related homeobox 2 |
| 5324 | PLAG1 | 8q12 | 13.72118649 | pleiomorphic adenoma gene 1 |
| 1602 | DACH1 | 13q22 | 14.3005626 | dachshund homolog 1 (Drosophila) |
| 122060 | SLAIN1 | 13q22.3 | 14.45670303 | SLAIN motif family, member 1 |
| 10221 | TRIB1 | 8q24.13 | 17.99469819 | tribbles homolog 1 (Drosophila) |
| 604 | BCL6 | 3q27 | 14.2017814 | B-cell CLL/lymphoma 6 |
| 6696 | SPP1 | 4q22.1 | 15.87480171 | secreted phosphoprotein 1 |
| 6659 | SOX4 | 6p22.3 | 23.61011778 | SRY (sex determining region Y)-box 4 |
| 56204 | KIAA1370 | 15q21.2-q21.3 | 12.95556474 | KIAA1370 |
| 81553 | FAM49A | 2p24.2 | 15.49789103 | family with sequence similarity 49, member A |
| 132864 | CPEB2 | 4p15.33 | 13.73070059 | cytoplasmic polyadenylation element binding protein 2 |
| 26575 | RGS17 | 6q25.3 | 15.19517797 | regulator of G-protein signaling 17 |
| 147906 | DACT3 | 19q13.32 | 17.2178528 | dapper, antagonist of beta-catenin, homolog 3 (Xenopus laevis) |
| 130367 | SGPP2 | 2q36.1 | 15.17894904 | sphingosine-1-phosphate phosphatase 2 |
| 5133 | PDCD1 | 2q37.3 | 13.150083 | programmed cell death 1 |
| 55803 | ADAP2 | 17q11.2 | 13.4232924 | ArfGAP with dual PH domains 2 |
| 7111 | TMOD1 | 9q22.3 | 15.86013706 | tropomodulin 1 |
| 27319 | BHLHE22 | 8q13 | 14.06625788 | basic helix-loop-helix family, member e22 |
| 10018 | BCL2L11 | 2q13 | 14.88719452 | BCL2-like 11 (apoptosis facilitator) |
| 125206 | SLC5A10 | 17p11.2 | 16.0780275 | solute carrier family 5 (sodium/glucose cotransporter), member 10 |
| 6899 | TBX1 | 22q11.21 | 12.18330237 | T-box 1 |
| 284161 | GDPD1 | 17q22 | 14.39892188 | glycerophosphodiester phosphodiesterase domain containing 1 |
| 2036 | EPB41L1 | 20q11.2-q12 | 13.42536018 | erythrocyte membrane protein band 4.1-like 1 |
| 79152 | FA2H | 16q23 | 12.70507805 | fatty acid 2-hydroxylase |
| 2259 | FGF14 | 13q34 | 15.9817524 | fibroblast growth factor 14 |
| 64219 | PJA1 | Xq13.1 | 13.87100102 | praja ring finger 1 |
| 9185 | REPS2 | Xp22.2 | 13.98578075 | RALBP1 associated Eps domain containing 2 |
| 213 | ALB | 4q13.3 | 12.45511698 | albumin |
| 57758 | SCUBE2 | 11p15.3 | 14.13630663 | signal peptide, CUB domain, EGF-like 2 |
| 5046 | PCSK6 | 15q26.3 | 11.97499961 | proprotein convertase subtilisin/kexin type 6 |
| 9619 | ABCG1 | 21q22.3 | 13.63307301 | ATP-binding cassette, sub-family G (WHITE), member 1 |
| 83473 | KATNAL2 | 18q21.1 | 16.36263662 | katanin p60 subunit A-like 2 |
| 1412 | CRYBA2 | 2q34-q36 | 11.66677032 | crystallin, beta A2 |
| 10149 | GPR64 | Xp22.13 | 26.22128834 | G protein-coupled receptor 64 |
| 11189 | CELF3 | 1q21 | 13.99242465 | CUGBP, Elav-like family member 3 |
| 3782 | KCNN3 | 1q21.3 | 11.69404264 | potassium intermediate/small conductance calcium-activated channel, subfamily N, member 3 |
| 2812 | GP1BB | 22q11.21-q11.23 | 11.21481046 | glycoprotein Ib (platelet), beta polypeptide |
| 79365 | BHLHE41 | 12p12.1 | 11.41611605 | basic helix-loop-helix family, member e41 |
| 10529 | NEBL | 10p12 | 11.53409842 | nebulette |
| 83850 | ESYT3 | 3q22.3 | 11.49817775 | extended synaptotagmin-like protein 3 |
| 1028 | CDKN1C | 11p15.5 | 11.42403184 | cyclin-dependent kinase inhibitor 1C (p57, Kip2) |
| 6236 | RRAD | 16q22 | 12.06146574 | Ras-related associated with diabetes |
| 284467 | FAM19A3 | 1p13.2 | 11.38747247 | family with sequence similarity 19 (chemokine (C-C motif)-like), member A3 |
| 257236 | CCDC96 | 4p16.1 | 14.90784687 | coiled-coil domain containing 96 |
| 57689 | LRRC4C | 11p12 | 12.05828223 | leucine rich repeat containing 4C |
| 28232 | SLCO3A1 | 15q26 | 11.4742928 | solute carrier organic anion transporter family, member 3A1 |
| 84553 | C6orf168 | 6q16.2 | 10.74554437 | chromosome 6 open reading frame 168 |
| 5251 | PHEX | Xp22.2-p22.1 | 11.26415094 | phosphate regulating endopeptidase homolog, X-linked |
| 9935 | MAFB | 20q11.2-q13.1 | 10.29829311 | v-maf musculoaponeurotic fibrosarcoma oncogene homolog B (avian) |
| 22996 | TTC39A | 1p32.3 | 18.35371239 | tetratricopeptide repeat domain 39A |
| 3485 | IGFBP2 | 2q33-q34 | 13.16506608 | insulin-like growth factor binding protein 2, 36kDa |
| 6096 | RORB | 9q22 | 15.51580524 | RAR-related orphan receptor B |
| 23209 | MLC1 | 22q13.33 | 10.32415998 | megalencephalic leukoencephalopathy with subcortical cysts 1 |
| 3848 | KRT1 | 12q12-q13 | 14.27038643 | keratin 1 |
| 11067 | C10orf10 | 10q11.21 | 11.57280489 | chromosome 10 open reading frame 10 |
| 27330 | RPS6KA6 | Xq21 | 12.09774848 | ribosomal protein S6 kinase, 90kDa, polypeptide 6 |
| 28984 | C13orf15 | 13q14.11 | 10.34718674 | chromosome 13 open reading frame 15 |
| 2308 | FOXO1 | 13q14.1 | 11.01535774 | forkhead box O1 |
| 9077 | DIRAS3 | 1p31 | 10.69847555 | DIRAS family, GTP-binding RAS-like 3 |
| 678 | ZFP36L2 | 2p22.3-p21 | 10.98161641 | zinc finger protein 36, C3H type-like 2 |
| 360 | AQP3 | 9p13 | 14.09879541 | aquaporin 3 (Gill blood group) |
| 114825 | PWWP2A | 5q33.3 | 10.83029023 | PWWP domain containing 2A |
| 1013 | CDH15 | 16q24.3 | 12.55914778 | cadherin 15, type 1, M-cadherin (myotubule) |
| 167691 | LCA5 | 6q14.1 | 11.19410024 | Leber congenital amaurosis 5 |
| 4312 | MMP1 | 11q22.3 | 12.99603834 | matrix metallopeptidase 1 (interstitial collagenase) |
| 26468 | LHX6 | 9q33.2 | 10.60495553 | LIM homeobox 6 |
| 85407 | NKD1 | 16q12 | 11.04554693 | naked cuticle homolog 1 (Drosophila) |
| 167681 | PRSS35 | 6q14.2 | 12.60566218 | protease, serine, 35 |
| 162494 | RHBDL3 | 17q11.2 | 10.97386631 | rhomboid, veinlet-like 3 (Drosophila) |
| 5522 | PPP2R2C | 4p16.1 | 10.69723969 | protein phosphatase 2, regulatory subunit B, gamma |
| 25960 | GPR124 | 8p11.23 | 10.46499705 | G protein-coupled receptor 124 |
| 4093 | SMAD9 | 13q12-q14 | 11.93124115 | SMAD family member 9 |
| 23105 | FSTL4 | 5q31.1 | 10.76507026 | follistatin-like 4 |
| 284427 | SLC25A41 | 19p13.3 | 10.24134529 | solute carrier family 25, member 41 |
| 5629 | PROX1 | 1q41 | 14.28768229 | prospero homeobox 1 |
| 23710 | GABARAPL1 | 12p13.2 | 9.384847704 | GABA(A) receptor-associated protein like 1 |
| 2681 | GGTA1 | 9q33.2 | 11.22258667 | glycoprotein, alpha-galactosyltransferase 1 pseudogene |
| 266722 | HS6ST3 | 13q32.1 | 12.13133711 | heparan sulfate 6-O-sulfotransferase 3 |
| 10675 | CSPG5 | 3p21.3 | 10.39964923 | chondroitin sulfate proteoglycan 5 (neuroglycan C) |
| 6615 | SNAI1 | 20q13.2 | 11.65330012 | snail homolog 1 (Drosophila) |
| 84870 | RSPO3 | 6q22.33 | 10.59393507 | R-spondin 3 homolog (Xenopus laevis) |
| 4047 | LSS | 21q22.3 | 9.294753898 | lanosterol synthase (2,3-oxidosqualene-lanosterol cyclase) |
| 112609 | MRAP2 | 6q14.2 | 10.50132889 | melanocortin 2 receptor accessory protein 2 |
| 6414 | SEPP1 | 5q31 | 9.472668078 | selenoprotein P, plasma, 1 |
| 8013 | NR4A3 | 9q22 | 14.09879541 | nuclear receptor subfamily 4, group A, member 3 |
| 49 | ACR | 22q13-qter | 10.15768686 | acrosin |
| 23186 | RCOR1 | 14q32.31 | 10.20669898 | REST corepressor 1 |
| 55175 | KLHL11 | 17q21.2 | 9.561034109 | kelch-like 11 (Drosophila) |
| 338382 | RAB7B | 1q32 | 11.97261731 | RAB7B, member RAS oncogene family |
| 201895 | C4orf34 | 4p14 | 9.058884022 | chromosome 4 open reading frame 34 |
| 84220 | RGPD5 | 2q13 | 8.992672944 | RANBP2-like and GRIP domain containing 5 |
| 285489 | DOK7 | 4p16.3 | 10.6589986 | docking protein 7 |
| 9413 | FAM189A2 | 9q21.11 | 9.277053534 | family with sequence similarity 189, member A2 |
| 7803 | PTP4A1 | 6q12 | 8.802791586 | protein tyrosine phosphatase type IVA, member 1 |
| 64762 | FAM59A | 18q12.1 | 9.317509885 | family with sequence similarity 59, member A |
| 10551 | AGR2 | 7p21.3 | 9.688903025 | anterior gradient homolog 2 (Xenopus laevis) |
| 63027 | SLC22A23 | 6p25.2 | 8.95724803 | solute carrier family 22, member 23 |
| 81631 | MAP1LC3B | 16q24.2 | 9.361026026 | microtubule-associated protein 1 light chain 3 beta |
| 57161 | PELI2 | 14q21 | 9.642006039 | pellino homolog 2 (Drosophila) |
| 1953 | MEGF6 | 1p36.3 | 9.713559075 | multiple EGF-like-domains 6 |
| 63973 | NEUROG2 | 4q25 | 13.02952841 | neurogenin 2 |
| 3209 | HOXA13 | 7p15.2 | 10.28877987 | homeobox A13 |
| 9537 | TP53I11 | 11p11.2 | 8.816598867 | tumor protein p53 inducible protein 11 |
| 7076 | TIMP1 | Xp11.3-p11.23 | 9.059194108 | TIMP metallopeptidase inhibitor 1 |
| 148398 | SAMD11 | 1p36.33 | 9.151447786 | sterile alpha motif domain containing 11 |
| 9148 | NEURL | 10q25.1 | 8.818296586 | neuralized homolog (Drosophila) |
| 9254 | CACNA2D2 | 3p21.3 | 10.26147821 | calcium channel, voltage-dependent, alpha 2/delta subunit 2 |
| 79844 | ZDHHC11 | 5p15.33 | 11.15150611 | zinc finger, DHHC-type containing 11 |
| 13 | AADAC | 3q21.3-q25.2 | 10.01312914 | arylacetamide deacetylase (esterase) |
| 2281 | FKBP1B | 2p23.3 | 8.6938789 | FK506 binding protein 1B, 12.6 kDa |
| 83546 | RTBDN | 19p12 | 9.393525154 | retbindin |
| 3726 | JUNB | 19p13.2 | 8.602961213 | jun B proto-oncogene |
| 4897 | NRCAM | 7q31 | 9.945197351 | neuronal cell adhesion molecule |
| 2774 | GNAL | 18p11.22-p11.21 | 9.009830717 | guanine nucleotide binding protein (G protein), alpha activating activity polypeptide, olfactory type |
| 389895 | LOC389895 | Xq27.1 | 9.374698042 | chromosome 16 open reading frame 72 pseudogene |
| 1960 | EGR3 | 8p23-p21 | 8.921790883 | early growth response 3 |
| 51207 | DUSP13 | 10q22.2 | 8.724061861 | dual specificity phosphatase 13 |
| 5733 | PTGER3 | 1p31.2 | 11.56975035 | prostaglandin E receptor 3 (subtype EP3) |
| 90024 | FLJ20021 | 4q24 | 8.257232531 | hypothetical LOC90024 |
| 92 | ACVR2A | 2q22.3 | 10.15416709 | activin A receptor, type IIA |
| 8325 | FZD8 | 10p11.21 | 9.551098469 | frizzled homolog 8 (Drosophila) |
| 8851 | CDK5R1 | 17q11.2 | 9.350217988 | cyclin-dependent kinase 5, regulatory subunit 1 (p35) |
| 23368 | PPP1R13B | 14q32.33 | 8.643805842 | protein phosphatase 1, regulatory (inhibitor) subunit 13B |
| 124404 | 12-Sep | 16p13.3 | 8.998388568 | septin 12 |
| 152006 | RNF38 | 9p13 | 8.677824042 | ring finger protein 38 |
| 1485 | CTAG1B | Xq28 | 9.942899788 | cancer/testis antigen 1B |
| 51083 | GAL | 11q13.3 | 9.970505612 | galanin prepropeptide |
| 55084 | SOBP | 6q21 | 11.08855909 | sine oculis binding protein homolog (Drosophila) |
| 23231 | SEL1L3 | 4p15.2 | 9.988952063 | sel-1 suppressor of lin-12-like 3 (C. elegans) |
| 10396 | ATP8A1 | 4p13 | 9.352378597 | ATPase, aminophospholipid transporter (APLT), class I, type 8A, member 1 |
| 6526 | SLC5A3 | 21q22.12 | 9.568768974 | solute carrier family 5 (sodium/myo-inositol cotransporter), member 3 |
| 113263 | GLCCI1 | 7p21.3 | 8.82645019 | glucocorticoid induced transcript 1 |
| 26022 | TMEM98 | 17q11.2 | 9.825290067 | transmembrane protein 98 |
| 9066 | SYT7 | 11q12-q13.1 | 8.829219012 | synaptotagmin VII |
| 1746 | DLX2 | 2q32 | 8.761769261 | distal-less homeobox 2 |
| 22983 | MAST1 | 19p13.2 | 8.874505094 | microtubule associated serine/threonine kinase 1 |
| 10633 | RASL10A | 22q12.2 | 9.29851286 | RAS-like, family 10, member A |
| 4325 | MMP16 | 8q21.3 | 9.018161418 | matrix metallopeptidase 16 (membrane-inserted) |
| 5334 | PLCL1 | 2q33 | 10.74182089 | phospholipase C-like 1 |
| 3624 | INHBA | 7p15-p13 | 10.95486651 | inhibin, beta A |
| 57458 | TMCC3 | 12q22 | 7.922742975 | transmembrane and coiled-coil domain family 3 |
| 8359 | HIST1H4A | 6p21.3 | 9.635325025 | histone cluster 1, H4a |
| 5168 | ENPP2 | 8q24.1 | 8.907372946 | ectonucleotide pyrophosphatase/phosphodiesterase 2 |
| 142891 | SAMD8 | 10q22.2 | 8.55044793 | sterile alpha motif domain containing 8 |
| 79605 | PGBD5 | 1q42.13 | 10.10501809 | piggyBac transposable element derived 5 |
| 2354 | FOSB | 19q13.32 | 12.07471027 | FBJ murine osteosarcoma viral oncogene homolog B |
| 54972 | TMEM132A | 11q12.2 | 7.953923391 | transmembrane protein 132A |
| 467 | ATF3 | 1q32.3 | 8.425590371 | activating transcription factor 3 |
| 6329 | SCN4A | 17q23.3 | 8.901160975 | sodium channel, voltage-gated, type IV, alpha subunit |
| 221687 | RNF182 | 6p23 | 8.28798977 | ring finger protein 182 |
| 80319 | CXXC4 | 4q22-q24 | 8.89503326 | CXXC finger protein 4 |
| 7067 | THRA | 17q11.2 | 8.050062588 | thyroid hormone receptor, alpha (erythroblastic leukemia viral (v-erb-a) oncogene homolog, avian) |
| 55531 | ELMOD1 | 11q22.3 | 9.22788484 | ELMO/CED-12 domain containing 1 |
| 1271 | CNTFR | 9p13 | 8.23034266 | ciliary neurotrophic factor receptor |
| 7164 | TPD52L1 | 6q22-q23 | 8.332022104 | tumor protein D52-like 1 |
| 57186 | RALGAPA2 | 20p11.22 | 7.869531653 | Ral GTPase activating protein, alpha subunit 2 (catalytic) |
| 84222 | TMEM191A | 22q11.21 | 8.370613317 | transmembrane protein 191A |
| 147798 | TMC4 | 19q13.42 | 7.966798025 | transmembrane channel-like 4 |
| 284600 | LOC284600 | 1p36.33 | 8 | hypothetical LOC284600 |
| 4129 | MAOB | Xp11.23 | 8.648800143 | monoamine oxidase B |
| 3775 | KCNK1 | 1q42-q43 | 7.904458683 | potassium channel, subfamily K, member 1 |
| 26270 | FBXO6 | 1p36.22 | 7.9465758 | F-box protein 6 |
| 1050 | CEBPA | 19q13.1 | 9.031715238 | CCAAT/enhancer binding protein (C/EBP), alpha |
| 401237 | FLJ22536 | 6p22.3 | 9.645348283 | hypothetical locus LOC401237 |
| 2035 | EPB41 | 1p33-p32 | 8.340689585 | erythrocyte membrane protein band 4.1 (elliptocytosis 1, RH-linked) |
| 8718 | TNFRSF25 | 1p36.2 | 9.277053534 | tumor necrosis factor receptor superfamily, member 25 |
| 158293 | FAM120AOS | 9q22.31 | 8.798961952 | family with sequence similarity 120A opposite strand |
| 5794 | PTPRH | 19q13.4 | 7.405856866 | protein tyrosine phosphatase, receptor type, H |
| 23433 | RHOQ | 2p21 | 8.410678693 | ras homolog gene family, member Q |
| 440224 | CXADRP3 | 18p11.21 | 8.603955124 | coxsackie virus and adenovirus receptor pseudogene 3 |
| 378805 | FLJ43663 | 7q32.3 | 8.233360986 | hypothetical LOC378805 |
| 2905 | GRIN2C | 17q25 | 9.380511985 | glutamate receptor, ionotropic, N-methyl D-aspartate 2C |
| 55605 | KIF21A | 12q12 | 7.680321303 | kinesin family member 21A |
| 440722 | LOC440722 | 1q42.12 | 8.029629012 | LOC440722 |
| 51130 | ASB3 | 2p16-p14 | 7.858932351 | ankyrin repeat and SOCS box containing 3 |
| 7025 | NR2F1 | 5q14 | 8.619275853 | nuclear receptor subfamily 2, group F, member 1 |
| 7462 | LAT2 | 7q11.23 | 7.75790262 | linker for activation of T cells family, member 2 |
| 3291 | HSD11B2 | 16q22 | 7.928236506 | hydroxysteroid (11-beta) dehydrogenase 2 |
| 29942 | PURG | 8p11 | 8.944494808 | purine-rich element binding protein G |
| 360030 | NANOGNB | 12p13.31 | 8.807097678 | NANOG neighbor homeobox |
| 6558 | SLC12A2 | 5q23.3 | 7.939234997 | solute carrier family 12 (sodium/potassium/chloride transporters), member 2 |
| 55502 | HES6 | 2q37.3 | 7.813668232 | hairy and enhancer of split 6 (Drosophila) |
| 51265 | CDKL3 | 5q31 | 7.376824943 | cyclin-dependent kinase-like 3 |
| 130399 | ACVR1C | 2q24.1 | 7.73999875 | activin A receptor, type IC |
| 6303 | SAT1 | Xp22.1 | 8.439553456 | spermidine/spermine N1-acetyltransferase 1 |
| 84440 | RAB11FIP4 | 17q11.2 | 9.001334398 | RAB11 family interacting protein 4 (class II) |
| 2258 | FGF13 | Xq26.3 | 9.297438716 | fibroblast growth factor 13 |
| 84215 | ZNF541 | 19q13.33 | 7.168041611 | zinc finger protein 541 |
| 1040 | CDS1 | 4q21.23 | 7.927320653 | CDP-diacylglycerol synthase (phosphatidate cytidylyltransferase) 1 |
| 80725 | SRCIN1 | 17q12 | 11.42667165 | SRC kinase signaling inhibitor 1 |
| 79158 | GNPTAB | 12q23.2 | 7.311073894 | N-acetylglucosamine-1-phosphate transferase, alpha and beta subunits |
| 83439 | TCF7L1 | 2p11.2 | 8.752327159 | transcription factor 7-like 1 (T-cell specific, HMG-box) |
| 5154 | PDGFA | 7p22 | 8.788802861 | platelet-derived growth factor alpha polypeptide |
| 55103 | RALGPS2 | 1q25.2 | 7.589474521 | Ral GEF with PH domain and SH3 binding motif 2 |
| 157562 | LOC157562 | 8q22.3 | 7.361690193 | hypothetical protein LOC157562 |
| 6604 | SMARCD3 | 7q35-q36 | 7.317411202 | SWI/SNF related, matrix associated, actin dependent regulator of chromatin, subfamily d, member 3 |
| 441455 | LOC441455 | 9q22.33 | 7.053470773 | makorin ring finger protein 1 pseudogene |
| 51195 | RAPGEFL1 | 17q21.1 | 7.388765456 | Rap guanine nucleotide exchange factor (GEF)-like 1 |
| 5563 | PRKAA2 | 1p31 | 7.394742958 | protein kinase, AMP-activated, alpha 2 catalytic subunit |
| 2487 | FRZB | 2qter | 7.686534673 | frizzled-related protein |
| 51201 | ZDHHC2 | 8p22 | 7.180909929 | zinc finger, DHHC-type containing 2 |
| 9456 | HOMER1 | 5q14.2 | 7.34451216 | homer homolog 1 (Drosophila) |
| 340719 | NANOS1 | 10q26.11 | 7.096680934 | nanos homolog 1 (Drosophila) |
| 9595 | CYTIP | 2q11.2 | 10.20198357 | cytohesin 1 interacting protein |
| 6474 | SHOX2 | 3q25.32 | 7.029067618 | short stature homeobox 2 |
| 150864 | FAM117B | 2q33.2 | 6.775527797 | family with sequence similarity 117, member B |
| 51308 | REEP2 | 5q31 | 8.104182521 | receptor accessory protein 2 |
| 389792 | IER5L | 9q34.11 | 7.563216934 | immediate early response 5-like |
| 55870 | ASH1L | 1q22 | 7.135428155 | ash1 (absent, small, or homeotic)-like (Drosophila) |
| 5362 | PLXNA2 | 1q32.2 | 7.180909929 | plexin A2 |
| 7432 | VIP | 6q25 | 7.797437047 | vasoactive intestinal peptide |
| 64061 | TSPYL2 | Xp11.2 | 7.495692285 | TSPY-like 2 |
| 23429 | RYBP | 3p13 | 7.71589405 | RING1 and YY1 binding protein |
| 9590 | AKAP12 | 6q24-q25 | 6.984816536 | A kinase (PRKA) anchor protein 12 |
| 150465 | TTL | 2q13 | 7.134329151 | tubulin tyrosine ligase |
| 1302 | COL11A2 | 6p21.3 | 7.225012081 | collagen, type XI, alpha 2 |
| 114569 | MAL2 | 8q23 | 8.359017223 | mal, T-cell differentiation protein 2 (gene/pseudogene) |
| 2261 | FGFR3 | 4p16.3 | 7.690087427 | fibroblast growth factor receptor 3 |
| 64121 | RRAGC | 1p34 | 6.810056023 | Ras-related GTP binding C |
| 30837 | SOCS7 | 17q12 | 7.252608633 | suppressor of cytokine signaling 7 |
| 5990 | RFX2 | 19p13.3-p13.2 | 7.428134818 | regulatory factor X, 2 (influences HLA class II expression) |
| 9480 | ONECUT2 | 18q21.31 | 7.05978868 | one cut homeobox 2 |
| 80821 | DDHD1 | 14q21 | 6.724840266 | DDHD domain containing 1 |
| 57571 | CARNS1 | 11q13.2 | 8.921790883 | carnosine synthase 1 |
| 2786 | GNG4 | 1q42.3 | 6.982127329 | guanine nucleotide binding protein (G protein), gamma 4 |
| 1820 | ARID3A | 19p13.3 | 6.999221401 | AT rich interactive domain 3A (BRIGHT-like) |
| 440455 | LOC440455 | 17q23.3 | 6.82817484 | hypothetical LOC440455 |
| 2778 | GNAS | 20q13.3 | 8.10980186 | GNAS complex locus |
| 5569 | PKIA | 8q21.12 | 6.975677454 | protein kinase (cAMP-dependent, catalytic) inhibitor alpha |
| 23097 | CDK19 | 6q21 | 6.390815332 | cyclin-dependent kinase 19 |
| 6324 | SCN1B | 19q13.1 | 6.857834492 | sodium channel, voltage-gated, type I, beta |
| 339456 | TMEM52 | 1p36.33 | 6.465073029 | transmembrane protein 52 |
| 7781 | SLC30A3 | 2p23.3 | 7.188379953 | solute carrier family 30 (zinc transporter), member 3 |
| 1525 | CXADR | 21q21.1 | 7.005288392 | coxsackie virus and adenovirus receptor |
| 1107 | CHD3 | 17p13.1 | 7.055100659 | chromodomain helicase DNA binding protein 3 |
| 3169 | FOXA1 | 14q12-q13 | 6.633789957 | forkhead box A1 |
| 80055 | PGAP1 | 2q33.1 | 6.424125156 | post-GPI attachment to proteins 1 |
| 286343 | C9orf150 | 9p23 | 6.537172046 | chromosome 9 open reading frame 150 |
| 92840 | REEP6 | 19p13.3 | 6.447172867 | receptor accessory protein 6 |
| 658 | BMPR1B | 4q22-q24 | 6.708067575 | bone morphogenetic protein receptor, type IB |
| 10846 | PDE10A | 6q26 | 6.606013876 | phosphodiesterase 10A |
| 5090 | PBX3 | 9q33.3 | 6.727171322 | pre-B-cell leukemia homeobox 3 |
| 85376 | RIMBP3 | 22q11.21 | 6.751306265 | RIMS binding protein 3 |
| 1809 | DPYSL3 | 5q32 | 7.126366427 | dihydropyrimidinase-like 3 |
| 56301 | SLC7A10 | 19q13.1 | 7.369953698 | solute carrier family 7, (neutral amino acid transporter, y+ system) member 10 |
| 51129 | ANGPTL4 | 19p13.3 | 6.411521088 | angiopoietin-like 4 |
| 6239 | RREB1 | 6p25 | 6.885206862 | ras responsive element binding protein 1 |
| 5778 | PTPN7 | 1q32.1 | 6.864331199 | protein tyrosine phosphatase, non-receptor type 7 |
| 9455 | HOMER2 | 15q24.3 | 6.520578659 | homer homolog 2 (Drosophila) |
| 23705 | CADM1 | 11q23.2 | 6.977289364 | cell adhesion molecule 1 |
| 1961 | EGR4 | 2p13 | 6.783359717 | early growth response 4 |
| 10912 | GADD45G | 9q22.1-q22.2 | 6.679159964 | growth arrest and DNA-damage-inducible, gamma |
| 122622 | ADSSL1 | 14q32.33 | 7.195026497 | adenylosuccinate synthase like 1 |
| 3363 | HTR7 | 10q21-q24 | 6.880082793 | 5-hydroxytryptamine (serotonin) receptor 7 (adenylate cyclase-coupled) |
| 80204 | FBXO11 | 2p16.3 | 6.439233156 | F-box protein 11 |
| 10006 | ABI1 | 10p11.2 | 7.13707698 | abl-interactor 1 |
| 23635 | SSBP2 | 5q14.1 | 7.580711879 | single-stranded DNA binding protein 2 |
| 114036 | C21orf82 | 21q22.11 | 6.978901646 | chromosome 21 open reading frame 82 |
| 53335 | BCL11A | 2p16.1 | 6.964672698 | B-cell CLL/lymphoma 11A (zinc finger protein) |
| 9314 | KLF4 | 9q31 | 7.125543205 | Kruppel-like factor 4 (gut) |
| 376267 | RAB15 | 14q23.3 | 7.104172765 | RAB15, member RAS onocogene family |
| 118611 | C10orf90 | 10q26.2 | 6.813203664 | chromosome 10 open reading frame 90 |
| 65986 | ZBTB10 | 8q13-q21.1 | 6.579093832 | zinc finger and BTB domain containing 10 |
| 2781 | GNAZ | 22q11.22 | 7.336032346 | guanine nucleotide binding protein (G protein), alpha z polypeptide |
| 83543 | AIF1L | 9q34.13-q34.3 | 6.411521088 | allograft inflammatory factor 1-like |
| 26249 | KLHL3 | 5q31 | 6.570485606 | kelch-like 3 (Drosophila) |
| 55636 | CHD7 | 8q12.2 | 6.389584957 | chromodomain helicase DNA binding protein 7 |
| 2817 | GPC1 | 2q35-q37 | 6.107082495 | glypican 1 |
| 3797 | KIF3C | 2p23 | 6.394507878 | kinesin family member 3C |
| 654 | BMP6 | 6p24-p23 | 6.386387091 | bone morphogenetic protein 6 |
| 6309 | SC5DL | 11q23.3 | 7.505769572 | sterol-C5-desaturase (ERG3 delta-5-desaturase homolog, S. cerevisiae)-like |
| 400966 | RGPD1 | 2p11.2 | 6.85745305 | RANBP2-like and GRIP domain containing 1 |
| 9182 | RASSF9 | 12q21.31 | 7.754830446 | Ras association (RalGDS/AF-6) domain family (N-terminal) member 9 |
| 170302 | ARX | Xp21 | 7.550994501 | aristaless related homeobox |
| 5187 | PER1 | 17p13.1 | 6.034217367 | period homolog 1 (Drosophila) |
| 57134 | MAN1C1 | 1p35 | 6.066369327 | mannosidase, alpha, class 1C, member 1 |
| 55321 | C20orf46 | 20p13 | 6.178828512 | chromosome 20 open reading frame 46 |
| 222389 | BEND7 | 10p13 | 7.514445588 | BEN domain containing 7 |
| 255809 | C19orf38 | 19p13.2 | 6.351829654 | chromosome 19 open reading frame 38 |
| 28514 | DLL1 | 6q27 | 7.143345988 | delta-like 1 (Drosophila) |
| 388308 | LOC388308 | 16q24.3 | 6.38786283 | similar to Brain-type organic cation transporter (Solute carrier family 22, member 17) |
| 10220 | GDF11 | 12q13.2 | 6.849506276 | growth differentiation factor 11 |
| 23414 | ZFPM2 | 8q23 | 6.441217166 | zinc finger protein, multitype 2 |
| 203429 | NCRNA00246 | Xq13.2 | 6.913102581 | non-protein coding RNA 246 |
| 57449 | PLEKHG5 | 1p36.31 | 6.6992519 | pleckstrin homology domain containing, family G (with RhoGef domain) member 5 |
| 6304 | SATB1 | 3p23 | 6.237638741 | SATB homeobox 1 |
| 5261 | PHKG2 | 16p11.2 | 6.09939719 | phosphorylase kinase, gamma 2 (testis) |
| 7546 | ZIC2 | 13q32 | 5.987691947 | Zic family member 2 (odd-paired homolog, Drosophila) |
| 285352 | FLJ39534 | 3p21.31 | 6.576560831 | hypothetical FLJ39534 |
| 389852 | SPACA5 | Xp11.23 | 6.427258042 | sperm acrosome associated 5 |
| 91694 | LONRF1 | 8p23.1 | 6.337512408 | LON peptidase N-terminal domain and ring finger 1 |
| 440944 | LOC440944 | 3p25.3 | 7.022724619 | hypothetical LOC440944 |
| 259282 | BOD1L | 4p16.1 | 5.974632601 | biorientation of chromosomes in cell division 1-like |
| 146330 | FBXL16 | 16p13.3 | 6.637622891 | F-box and leucine-rich repeat protein 16 |
| 6496 | SIX3 | 2p21 | 6.424053004 | SIX homeobox 3 |
| 5153 | PDE1B | 12q13 | 6.139055305 | phosphodiesterase 1B, calmodulin-dependent |
| 51274 | KLF3 | 4p14 | 5.95595698 | Kruppel-like factor 3 (basic) |
| 55294 | FBXW7 | 4q31.3 | 6.041890342 | F-box and WD repeat domain containing 7 |
| 22881 | ANKRD6 | 6q14.2-q16.1 | 5.729198538 | ankyrin repeat domain 6 |
| 143098 | MPP7 | 10p12.1 | 6.103626436 | membrane protein, palmitoylated 7 (MAGUK p55 subfamily member 7) |
| 9648 | GCC2 | 2q12.3 | 6.279936625 | GRIP and coiled-coil domain containing 2 |
| 27134 | TJP3 | 19p13.3 | 6.541704839 | tight junction protein 3 (zona occludens 3) |
| 29993 | PACSIN1 | 6p21.3 | 5.868533377 | protein kinase C and casein kinase substrate in neurons 1 |
| 4642 | MYO1D | 17q11-q12 | 6.875668552 | myosin ID |
| 1296 | COL8A2 | 1p34.2 | 7.089415402 | collagen, type VIII, alpha 2 |
| 9625 | AATK | 17q25.3 | 6.97782675 | apoptosis-associated tyrosine kinase |
| 10458 | BAIAP2 | 17q25 | 5.904916532 | BAI1-associated protein 2 |
| 7802 | DNALI1 | 1p35.1 | 6.835277927 | dynein, axonemal, light intermediate chain 1 |
| 79956 | ERMP1 | 9p24 | 5.859049614 | endoplasmic reticulum metallopeptidase 1 |
| 9767 | PHF16 | Xp11.23 | 5.656854249 | PHD finger protein 16 |
| 5874 | RAB27B | 18q21.2 | 5.770381352 | RAB27B, member RAS oncogene family |
| 6498 | SKIL | 3q26 | 6.490516699 | SKI-like oncogene |
| 388403 | YPEL2 | 17q22 | 6.139457204 | yippee-like 2 (Drosophila) |
| 7038 | TG | 8q24 | 5.798447451 | thyroglobulin |
| 85477 | SCIN | 7p21.3 | 6.086727028 | scinderin |
| 129446 | XIRP2 | 2q24.3 | 6.081104297 | xin actin-binding repeat containing 2 |
| 7408 | VASP | 19q13.2-q13.3 | 5.818981529 | vasodilator-stimulated phosphoprotein |
| 22846 | VASH1 | 14q24.3 | 6.124109166 | vasohibin 1 |
| 171024 | SYNPO2 | 4q26 | 8.097631547 | synaptopodin 2 |
| 200424 | TET3 | 2p13.1 | 5.815890069 | tet oncogene family member 3 |
| 138151 | NACC2 | 9q34.3 | 6.716299969 | NACC family member 2, BEN and BTB (POZ) domain containing |
| 339983 | NAT8L | 4p16.3 | 6.230436899 | N-acetyltransferase 8-like (GCN5-related, putative) |
| 441428 | LOC441428 | 9p11.2 | 5.779053931 | hypothetical gene supported by BX641014 |
| 57149 | LYRM1 | 16p11.2 | 6.259294254 | LYR motif containing 1 |
| 57662 | KIAA1543 | 19p13.2 | 6.311623336 | KIAA1543 |
| 3714 | JAG2 | 14q32 | 6.568967677 | jagged 2 |
| 58508 | MLL3 | 7q36.1 | 5.792778718 | myeloid/lymphoid or mixed-lineage leukemia 3 |
| 55231 | CCDC87 | 11q13.2 | 5.920285158 | coiled-coil domain containing 87 |
| 56987 | BBX | 3q13.1 | 5.642495385 | bobby sox homolog (Drosophila) |
| 64083 | GOLPH3 | 5p13.3 | 5.505346367 | golgi phosphoprotein 3 (coat-protein) |
| 29841 | GRHL1 | 2p25.1 | 6.276672783 | grainyhead-like 1 (Drosophila) |
| 51696 | HECA | 6q23-q24 | 5.534041003 | headcase homolog (Drosophila) |
| 7172 | TPMT | 6p22.3 | 5.866725763 | thiopurine S-methyltransferase |
| 687 | KLF9 | 9q13 | 5.964219415 | Kruppel-like factor 9 |
| 81928 | CABLES2 | 20q13.33 | 5.685681692 | Cdk5 and Abl enzyme substrate 2 |
| 55603 | FAM46A | 6q14 | 5.531484324 | family with sequence similarity 46, member A |
| 59335 | PRDM12 | 9q33-q34 | 5.87395956 | PR domain containing 12 |
| 22849 | CPEB3 | 10q23.32 | 6.604731897 | cytoplasmic polyadenylation element binding protein 3 |
| 8740 | TNFSF14 | 19p13.3 | 6.232344577 | tumor necrosis factor (ligand) superfamily, member 14 |
| 5971 | RELB | 19q13.32 | 5.474270281 | v-rel reticuloendotheliosis viral oncogene homolog B |
| 151195 | CCNYL1 | 2q33.3 | 6.362821576 | cyclin Y-like 1 |
| 364 | AQP7 | 9p13 | 6.308658834 | aquaporin 7 |
| 92162 | TMEM88 | 17p13.1 | 5.671249654 | transmembrane protein 88 |
| 285172 | FAM126B | 2q33.1 | 6.000155957 | family with sequence similarity 126, member B |
| 51523 | CXXC5 | 5q31.2 | 5.684368173 | CXXC finger protein 5 |
| 54438 | GFOD1 | 6pter-p22.1 | 5.703956435 | glucose-fructose oxidoreductase domain containing 1 |
| 4783 | NFIL3 | 9q22 | 5.392172561 | nuclear factor, interleukin 3 regulated |
| 23287 | AGTPBP1 | 9q21.33 | 5.790414665 | ATP/GTP binding protein 1 |
| 158584 | FAAH2 | Xp11.21 | 5.994613197 | fatty acid amide hydrolase 2 |
| 9990 | SLC12A6 | 15q13 | 5.54299869 | solute carrier family 12 (potassium/chloride transporters), member 6 |
| 7089 | TLE2 | 19p13.3 | 5.33442708 | transducin-like enhancer of split 2 (E(sp1) homolog, Drosophila) |
| 152 | ADRA2C | 4p16 | 5.751746131 | adrenergic, alpha-2C-, receptor |
| 9363 | RAB33A | Xq26.1 | 6.689971199 | RAB33A, member RAS oncogene family |
| 51481 | VCX3A | Xp22 | 6.067070181 | variable charge, X-linked 3A |
| 84796 | MGC13053 | 11q23.3 | 6.555592982 | hypothetical MGC13053 |
| 148979 | GLIS1 | 1p32.3 | 7.181739548 | GLIS family zinc finger 1 |
| 79623 | GALNT14 | 2p23.1 | 6.730280654 | UDP-N-acetyl-alpha-D-galactosamine:polypeptide N-acetylgalactosaminyltransferase 14 (GalNAc-T14) |
| 150166 | LOC150166 | 22q11.21 | 6.294099563 | hypothetical protein LOC150166 |
| 2737 | GLI3 | 7p13 | 5.761056207 | GLI family zinc finger 3 |
| 23412 | COMMD3 | 10pter-q22.1 | 5.274776624 | COMM domain containing 3 |
| 1396 | CRIP1 | 14q32.33 | 5.65554739 | cysteine-rich protein 1 (intestinal) |
| 284804 | LOC284804 | 20q11.21 | 5.452810429 | hypothetical protein LOC284804 |
| 220001 | VWCE | 11q12.2 | 5.445256466 | von Willebrand factor C and EGF domains |
| 11043 | MID2 | Xq22.3 | 5.718618511 | midline 2 |
| 147699 | PPM1N | 19q13.32 | 5.58671413 | protein phosphatase, Mg2+/Mn2+ dependent, 1N (putative) |
| 55279 | ZNF654 | 3p11.1 | 5.984114491 | zinc finger protein 654 |
| 5744 | PTHLH | 12p12.1-p11.2 | 5.729198538 | parathyroid hormone-like hormone |
| 375484 | C5orf25 | 5q35.2 | 5.190955068 | chromosome 5 open reading frame 25 |
| 93099 | DMKN | 19q13.12 | 6.137566141 | dermokine |
| 4291 | MLF1 | 3q25.1 | 5.570602344 | myeloid leukemia factor 1 |
| 5818 | PVRL1 | 11q23.3 | 5.450920956 | poliovirus receptor-related 1 (herpesvirus entry mediator C) |
| 11177 | BAZ1A | 14q13.2 | 5.229476989 | bromodomain adjacent to zinc finger domain, 1A |
| 56967 | C14orf132 | 14q32.2 | 5.553022141 | chromosome 14 open reading frame 132 |
| 126567 | C2CD4C | 19p13.3 | 5.375255507 | C2 calcium-dependent domain containing 4C |
| 146223 | CMTM4 | 16q21-q22.1 | 5.81723398 | CKLF-like MARVEL transmembrane domain containing 4 |
| 81558 | FAM117A | 17q21.33 | 5.718618511 | family with sequence similarity 117, member A |
| 148252 | DIRAS1 | 19p13.3 | 6.136148226 | DIRAS family, GTP-binding RAS-like 1 |
| 23242 | COBL | 7p12.1 | 6.64569348 | cordon-bleu homolog (mouse) |
| 2776 | GNAQ | 9q21 | 6.279573892 | guanine nucleotide binding protein (G protein), q polypeptide |
| 84777 | MGC11082 | 18p11.31 | 5.40459003 | hypothetical LOC84777 |
| 8621 | CDK13 | 7p13 | 5.279251268 | cyclin-dependent kinase 13 |
| 7779 | SLC30A1 | 1q32.3 | 6.15034213 | solute carrier family 30 (zinc transporter), member 1 |
| 27345 | KCNMB4 | 12q | 7.079594194 | potassium large conductance calcium-activated channel, subfamily M, beta member 4 |
| 60370 | AVPI1 | 10q24.2 | 5.199357418 | arginine vasopressin-induced 1 |
| 23528 | ZNF281 | 1q32.1 | 5.355421028 | zinc finger protein 281 |
| 160622 | GRASP | 12q13.13 | 5.680429434 | GRP1 (general receptor for phosphoinositides 1)-associated scaffold protein |
| 4137 | MAPT | 17q21.1 | 5.673652448 | microtubule-associated protein tau |
| 29995 | LMCD1 | 3p26-p24 | 5.622973834 | LIM and cysteine-rich domains 1 |
| 29950 | SERTAD1 | 19q13.1-q13.2 | 5.414853186 | SERTA domain containing 1 |
| 220213 | OTUD1 | 10p12.2 | 5.19755577 | OTU domain containing 1 |
| 84837 | C14orf128 | 14q12 | 5.305418901 | chromosome 14 open reading frame 128 |
| 168448 | CDC14C | 7p12.3 | 5.431434702 | CDC14 cell division cycle 14 homolog C (S. cerevisiae) |
| 4293 | MAP3K9 | 14q24.3-q31 | 5.460374872 | mitogen-activated protein kinase kinase kinase 9 |
| 22927 | HABP4 | 9q22.3-q31 | 5.422657307 | hyaluronan binding protein 4 |
| 2887 | GRB10 | 7p12.2 | 6.455370947 | growth factor receptor-bound protein 10 |
| 10265 | IRX5 | 16q12.2 | 5.186758986 | iroquois homeobox 5 |
| 285 | ANGPT2 | 8p23.1 | 6.509595512 | angiopoietin 2 |
| 27075 | TSPAN13 | 7p21.1 | 5.208976758 | tetraspanin 13 |
| 57600 | FNIP2 | 4q32.1 | 5.171203214 | folliculin interacting protein 2 |
| 55132 | LARP1B | 4q28.2 | 5.378775511 | La ribonucleoprotein domain family, member 1B |
| 123606 | NIPA1 | 15q11.2 | 5.227664904 | non imprinted in Prader-Willi/Angelman syndrome 1 |
| 490 | ATP2B1 | 12q21.3 | 5.030377489 | ATPase, Ca++ transporting, plasma membrane 1 |
| 7328 | UBE2H | 7q32 | 5.463529819 | ubiquitin-conjugating enzyme E2H (UBC8 homolog, yeast) |
| 84954 | MPND | 19p13.3 | 5.320886636 | MPN domain containing |
| 63035 | BCORL1 | Xq25-q26.1 | 5.431434702 | BCL6 corepressor-like 1 |
| 57674 | RNF213 | 17q25.3 | 5.510224547 | ring finger protein 213 |
| 5270 | SERPINE2 | 2q33-q35 | 5.315971365 | serpin peptidase inhibitor, clade E (nexin, plasminogen activator inhibitor type 1), member 2 |
| 5731 | PTGER1 | 19p13.1 | 5.401911484 | prostaglandin E receptor 1 (subtype EP1), 42kDa |
| 10905 | MAN1A2 | 1p13 | 5.046286869 | mannosidase, alpha, class 1A, member 2 |
| 2332 | FMR1 | Xq27.3 | 5.303090369 | fragile X mental retardation 1 |
| 9047 | SH2D2A | 1q21 | 5.073565597 | SH2 domain containing 2A |
| 2077 | ERF | 19q13 | 5.077083544 | Ets2 repressor factor |
| 7093 | TLL2 | 10q23-q24 | 6.893165561 | tolloid-like 2 |
| 8976 | WASL | 7q31.3 | 5.225853448 | Wiskott-Aldrich syndrome-like |
| 124976 | SPNS2 | 17p13.2 | 6.208163988 | spinster homolog 2 (Drosophila) |
| 196515 | FLJ30092 | 12q24.13 | 5.395578978 | AF-1 specific protein phosphatase |
| 8320 | EOMES | 3p24.1 | 16.14302356 | eomesodermin |
| 659 | BMPR2 | 2q33-q34 | 5.206570256 | bone morphogenetic protein receptor, type II (serine/threonine kinase) |
| 55624 | POMGNT1 | 1p34.1 | 5.137629228 | protein O-linked mannose beta1,2-N-acetylglucosaminyltransferase |
| 6939 | TCF15 | 20p13 | 6.687653034 | transcription factor 15 (basic helix-loop-helix) |
| 154807 | VKORC1L1 | 7q11.21 | 4.978346566 | vitamin K epoxide reductase complex, subunit 1-like 1 |
| 158046 | NXNL2 | 9q22.1 | 5.301078226 | nucleoredoxin-like 2 |
| 85455 | DISP2 | 15q15.1 | 5.536598864 | dispatched homolog 2 (Drosophila) |
| 27439 | CECR6 |  | 5.469213318 | cat eye syndrome chromosome region, candidate 6 |
| 84628 | NTNG2 | 9q34 | 5.419025115 | netrin G2 |
| 2296 | FOXC1 | 6p25 | 6.564668782 | forkhead box C1 |
| 80301 | PLEKHO2 | 15q22.1 | 5.3665689 | pleckstrin homology domain containing, family O member 2 |
| 27092 | CACNG4 | 17q24 | 5.073565597 | calcium channel, voltage-dependent, gamma subunit 4 |
| 6256 | RXRA | 9q34.3 | 5.227061016 | retinoid X receptor, alpha |
| 64753 | CCDC136 | 7q33 | 5.727874965 | coiled-coil domain containing 136 |
| 29944 | PNMA3 | Xq28 | 5.77705141 | paraneoplastic antigen MA3 |
| 145407 | C14orf37 | 14q23.1 | 5.022248217 | chromosome 14 open reading frame 37 |
| 114990 | VASN | 16p13.3 | 5.25127106 | vasorin |
| 9832 | JAKMIP2 | 5q32 | 5.187458097 | janus kinase and microtubule interacting protein 2 |
| 124783 | C17orf46 | 17q21.31 | 5.245208057 | chromosome 17 open reading frame 46 |
| 55876 | GSDMB | 17q12 | 5.144389934 | gasdermin B |
| 93145 | OLFM2 | 19p13.2 | 5.566099377 | olfactomedin 2 |
| 286336 | FAM78A | 9q34 | 5.380432781 | family with sequence similarity 78, member A |
| 153514 | C14orf81 | 14q32.33 | 4.862393551 | chromosome 14 open reading frame 81 |
| 145226 | RDH12 | 14q24.1 | 4.852292994 | retinol dehydrogenase 12 (all-trans/9-cis/11-cis) |
| 80318 | GKAP1 | 9q21.32 | 4.824490338 | G kinase anchoring protein 1 |
| 51320 | MEX3C | 18q21.2 | 5.423283793 | mex-3 homolog C (C. elegans) |
| 9423 | NTN1 | 17p13-p12 | 5.333019106 | netrin 1 |
| 55964 | 3-Sep | 22q13.2 | 5.383956175 | septin 3 |
| 3757 | KCNH2 | 7q36.1 | 6.821079134 | potassium voltage-gated channel, subfamily H (eag-related), member 2 |
| 6095 | RORA | 15q22.2 | 5.856342784 | RAR-related orphan receptor A |
| 387893 | SETD8 | 12q24.31 | 4.791390653 | SET domain containing (lysine methyltransferase) 8 |
| 222166 | C7orf41 | 7p14.3 | 5.047841695 | chromosome 7 open reading frame 41 |
| 5600 | MAPK11 | 22q13.33 | 4.946243737 | mitogen-activated protein kinase 11 |
| 400756 | LOC400756 | 1p31.3 | 5.234312286 | hypothetical LOC400756 |
| 8850 | KAT2B | 3p24 | 4.961122796 | K(lysine) acetyltransferase 2B |
| 2306 | FOXD2 | 1p34-p32 | 5.260378708 | forkhead box D2 |
| 4130 | MAP1A | 15q15.3 | 5.400713435 | microtubule-associated protein 1A |
| 10156 | RASA4 | 7q22 | 5.000826895 | RAS p21 protein activator 4 |
| 1907 | EDN2 | 1p34 | 6.224681412 | endothelin 2 |
| 60468 | BACH2 | 6q15 | 5.432689775 | BTB and CNC homology 1, basic leucine zipper transcription factor 2 |
| 4953 | ODC1 | 2p25 | 4.755729526 | ornithine decarboxylase 1 |
| 148046 | C19orf23 | 19p13.3 | 5.486792295 | chromosome 19 open reading frame 23 |
| 7077 | TIMP2 | 17q25 | 4.76708825 | TIMP metallopeptidase inhibitor 2 |
| 181 | AGRP | 16q22 | 5.205367422 | agouti related protein homolog (mouse) |
| 3764 | KCNJ8 | 12p11.23 | 5.177778801 | potassium inwardly-rectifying channel, subfamily J, member 8 |
| 89780 | WNT3A | 1q42 | 5.942211683 | wingless-type MMTV integration site family, member 3A |
| 147968 | CAPN12 | 19q13.2 | 4.881529784 | calpain 12 |
| 153222 | C5orf41 | 5q35.1 | 5.452810429 | chromosome 5 open reading frame 41 |
| 10788 | IQGAP2 | 5q13.3 | 5.841477555 | IQ motif containing GTPase activating protein 2 |
| 440040 | LOC440040 | 11p11.12 | 4.962105802 | glutamate receptor, metabotropic 5 pseudogene |
| 7975 | MAFK | 7p22.3 | 4.941674549 | v-maf musculoaponeurotic fibrosarcoma oncogene homolog K (avian) |
| 2055 | CLN8 | 8p23 | 5.602225284 | ceroid-lipofuscinosis, neuronal 8 (epilepsy, progressive with mental retardation) |
| 445 | ASS1 | 9q34.1 | 4.860147168 | argininosuccinate synthase 1 |
| 898 | CCNE1 | 19q12 | 4.971266114 | cyclin E1 |
| 22990 | PCNX | 14q24.2 | 4.959976665 | pecanex homolog (Drosophila) |
| 57620 | STIM2 | 4p15.2 | 5.004872558 | stromal interaction molecule 2 |
| 51804 | SIX4 | 14q23 | 4.980072229 | SIX homeobox 4 |
| 124359 | CDYL2 | 16q23.2 | 4.883786051 | chromodomain protein, Y-like 2 |
| 4010 | LMX1B | 9q34 | 5.331648484 | LIM homeobox transcription factor 1, beta |
| 285603 | FLJ39485 | 5q15 | 4.979496942 | hypothetical protein FLJ39485 |
| 9223 | MAGI1 | 3p14.1 | 5.247632418 | membrane associated guanylate kinase, WW and PDZ domain containing 1 |
| 55529 | TMEM55A | 8q21.3 | 4.660010913 | transmembrane protein 55A |
| 3679 | ITGA7 | 12q13 | 5.192754427 | integrin, alpha 7 |
| 1999 | ELF3 | 1q32.2 | 5.834059096 | E74-like factor 3 (ets domain transcription factor, epithelial-specific ) |
| 55219 | TMEM57 | 1p36.11 | 4.735992074 | transmembrane protein 57 |
| 84735 | CNDP1 | 18q22.3 | 4.949753272 | carnosine dipeptidase 1 (metallopeptidase M20 family) |
| 10344 | CCL26 | 7q11.23 | 5.055428334 | chemokine (C-C motif) ligand 26 |
| 81571 | NCRNA00287 | 9q33.3 | 5.115942325 | non-protein coding RNA 287 |
| 374 | AREG | 4q13-q21 | 5.233103043 | amphiregulin |
| 57568 | SIPA1L2 | 1q42.2 | 4.931409302 | signal-induced proliferation-associated 1 like 2 |
| 81617 | CAB39L | 13q14.2 | 4.642815658 | calcium binding protein 39-like |
| 4216 | MAP3K4 | 6q26 | 4.719606787 | mitogen-activated protein kinase kinase kinase 4 |
| 22871 | NLGN1 | 3q26.31 | 5.028053498 | neuroligin 1 |
| 50861 | STMN3 | 20q13.3 | 4.791944208 | stathmin-like 3 |
| 116448 | OLIG1 | 21q22.11 | 5.33442708 | oligodendrocyte transcription factor 1 |
| 283209 | PGM2L1 | 11q13.4 | 4.832713044 | phosphoglucomutase 2-like 1 |
| 54 | ACP5 | 19p13.3-p13.2 | 5.320886636 | acid phosphatase 5, tartrate resistant |
