# Supplementary material for: Suppression of Breast Tumor Growth and Metastasis by an Engineered Transcription Factor
Source: PLoS One. 2011 Sep 13;6(9):e24595. doi: 10.1371/journal.pone.0024595 (PMC3172243; doi:10.1371/journal.pone.0024595)
Supplement: Table S2 — Pathway analysis of the 550-gene signature using the David database ( http://david.abcc.ncifcrf.gov/ ). (DOC) [file pone.0024595.s006.doc]

**Table S2. Pathway analysis of the 550-gene signature using the David database (http://david.abcc.ncifcrf.gov/)**

| **Pathways** | **p=Value** |
| --- | --- |
| Calcium signaling pathway | 1.50E-03 |
| Tight junction | 3.30E-03 |
| TGF-beta signaling pathway | 5.40E-03 |
| Neuroactive ligand-receptor interaction | 7.70E-03 |
| Arrhythmogenic right ventricular cardiomyopathy (ARVC) | 1.30E-02 |
| Regulation of actin cytoskeleton | 4.70E-02 |
| Notch signaling pathway | 5.70E-02 |
| Vascular smooth muscle contraction | 5.80E-02 |
| Pathways in cancer | 8.40E-02 |
| Vibrio cholerae infection | 8.60E-02 |
| Cell adhesion molecules (CAMs) | 1.00E-01 |
