# Supplementary material for: Suppression of Breast Tumor Growth and Metastasis by an Engineered Transcription Factor
Source: PLoS One. 2011 Sep 13;6(9):e24595. doi: 10.1371/journal.pone.0024595 (PMC3172243; doi:10.1371/journal.pone.0024595)
Supplement: Table S3 — Antibodies used in this study. (DOCX) [file pone.0024595.s007.docx]

**Table S3.** *Genes regulated with the Maspin cDNA*

| **Gene ID** | **NAME** | **Description** | **Fold change** |
| --- | --- | --- | --- |
| 5268 | SERPINB5 | Serpin peptidase inhibitor, clade B (ovalbumin), member 5 | 5.4075 |
| 57452 | GALNTL1 | UDP-N-acetyl-alpha-D-galactosamine:polypeptide N-acetylgalactosaminyltransferase-like 1 | 4.39 |
| 57535 | KIAA1324 | KIAA1324 | 3.883 |
| 27033 | ZBTB32 | Zinc finger and BTB domain containing 32 | 2.772333333 |
| 196385 | DNAH10 | Dynein, axonemal, heavy chain 10 | 2.471 |
| 205860 | FLJ25801 | Hypothetical protein FLJ25801 | 2.350333333 |
| 5655 | KLK10 | Kallikrein-related peptidase 10 | 2.316666667 |
| 374403 | TBC1D10C | TBC1 domain family, member 10C | 2.128666667 |
| 3560 | IL2RB | Interleukin 2 receptor, beta | 2.115 |
| 57571 | KIAA1394 | KIAA1394 protein | 2.111 |
| 1043 | CD52 | CD52 molecule | 2.044 |
| 6543 | SLC8A2 | Solute carrier family 8 (sodium-calcium exchanger), member 2 | 2.025 |
| 2819 | GPD1 | Glycerol-3-phosphate dehydrogenase 1 (soluble) | 1.954666667 |
| 84063 | KIRREL2 | Kin of IRRE like 2 (Drosophila) | 1.792 |
| 23475 | QPRT | Quinolinate phosphoribosyltransferase (nicotinate-nucleotide pyrophosphorylase (carboxylating)) | 1.624333333 |
| 3699 | ITIH3 | Inter-alpha (globulin) inhibitor H3 | 1.546 |
| 10148 | EBI3 | Epstein-Barr virus induced gene 3 | 1.390666667 |
| 94 | ACVRL1 | Activin A receptor type II-like 1 | 1.323666667 |
| 1907 | EDN2 | Endothelin 2 | 1.299333333 |
| 3552 | IL1A | Interleukin 1, alpha | 1.28 |
| 440503 | LSDP5 | Lipid storage droplet protein 5 | 1.253666667 |
| 387700 | SLC16A12 | Solute carrier family 16, member 12 (monocarboxylic acid transporter 12) | 1.172333333 |
| 716 | C1S | Complement component 1, s subcomponent | 1.163666667 |
| 9687 | GREB1 | GREB1 protein | 1.150666667 |
| 1958 | EGR1 | Early growth response 1 | 1.128666667 |
| 634 | CEACAM1 | Carcinoembryonic antigen-related cell adhesion molecule 1 (biliary glycoprotein) | 1.087666667 |
| 18 | ABAT | 4-aminobutyrate aminotransferase | 1.073 |
| 10148 | EBI3 | Epstein-Barr virus induced gene 3 | 1.039333333 |
| 2793 | GNGT2 | Guanine nucleotide binding protein (G protein), gamma transducing activity polypeptide 2 | 1.024666667 |
| 9625 | AATK | Apoptosis-associated tyrosine kinase | 1.016333333 |
| 623 | BDKRB1 | Bradykinin receptor B1 | 1.0125 |
| 219539 | YPEL4 | Yippee-like 4 (Drosophila) | 1.011 |
| 3718 | JAK3 | Janus kinase 3 (a protein tyrosine kinase, leukocyte) | 1.008333333 |
| 116844 | LRG1 | Leucine-rich alpha-2-glycoprotein 1 | 0.953 |
| 162494 | RHBDL3 | Rhomboid, veinlet-like 3 (Drosophila) | 0.893 |
| 8605 | PLA2G4C | Phospholipase A2, group IVC (cytosolic, calcium-independent) | 0.831333333 |
| 3604 | TNFRSF9 | Tumor necrosis factor receptor superfamily, member 9 | 0.821 |
| 147906 | DACT3 | Dapper, antagonist of beta-catenin, homolog 3 (Xenopus laevis) | 0.791 |
| 29993 | PACSIN1 | Protein kinase C and casein kinase substrate in neurons 1 | 0.782666667 |
| 6352 | CCL5 | Chemokine (C-C motif) ligand 5 | 0.781666667 |
| 7850 | IL1R2 | Interleukin 1 receptor, type II | 0.757 |
| 623 | BDKRB1 | Bradykinin receptor B1 | 0.742333333 |
| 282969 | C10orf125 | Chromosome 10 open reading frame 125 | 0.735333333 |
| 282969 | C10orf125 | Chromosome 10 open reading frame 125 | 0.727333333 |
| 126567 | FAM148C | Family with sequence similarity 148, member C | 0.7195 |
| 2786 | GNG4 | Guanine nucleotide binding protein (G protein), gamma 4 | 0.697 |
| 55466 | DNAJA4 | DnaJ (Hsp40) homolog, subfamily A, member 4 | 0.685 |
| 80115 | BAIAP2L2 | BAI1-associated protein 2-like 2 | 0.662 |
| 91543 | RSAD2 | Radical S-adenosyl methionine domain containing 2 | 0.639333333 |
| 26508 | HEYL | Hairy/enhancer-of-split related with YRPW motif-like | 0.633 |
| 25900 | HOM-TES-103 | Hypothetical protein LOC25900 | 0.616 |
| 25884 | CHRDL2 | Chordin-like 2 | 0.610333333 |
| 1013 | CDH15 | Cadherin 15, M-cadherin (myotubule) | 0.607666667 |
| 80307 | FER1L4 | Fer-1-like 4 (C. elegans) | 0.568666667 |
| 64699 | TMPRSS3 | Transmembrane protease, serine 3 | 0.560333333 |
| 221002 | RASGEF1A | RasGEF domain family, member 1A | 0.556 |
| 1949 | EFNB3 | Ephrin-B3 | 0.555333333 |
| 80725 | SNIP | SNAP25-interacting protein | 0.552 |
| 55287 | TMEM40 | Transmembrane protein 40 | 0.542333333 |
| 54544 | CRCT1 | Cysteine-rich C-terminal 1 | 0.536 |
| 53820 | DSCR6 | Down syndrome critical region gene 6 | 0.535 |
| 9148 | NEURL | Neuralized homolog (Drosophila) | 0.533 |
| 285489 | DOK7 | Docking protein 7 | 0.493 |
| 6775 | STAT4 | Signal transducer and activator of transcription 4 | 0.486333333 |
| 79933 | SYNPO2L | Synaptopodin 2-like | 0.472 |
| 53405 | CLIC5 | Chloride intracellular channel 5 | 0.46 |
| 93082 | LINCR | Likely ortholog of mouse lung-inducible Neutralized-related C3HC4 RING domain protein | 0.444666667 |
| 6319 | SCD | Stearoyl-CoA desaturase (delta-9-desaturase) | 0.437333333 |
| 6615 | SNAI1 | Snail homolog 1 (Drosophila) | 0.422666667 |
| 2686 | GGTL3 | Gamma-glutamyltransferase-like 3 | 0.402 |
| 7031 | TFF1 | Trefoil factor 1 | 0.400666667 |
| 10158 | PDZK1IP1 | PDZK1 interacting protein 1 | 0.354333333 |
| 6273 | S100A2 | S100 calcium binding protein A2 | 0.349 |
| 51466 | EVL | Enah/Vasp-like | 0.330333333 |
| 3726 | JUNB | Jun B proto-oncogene | 0.324 |
| 3553 | IL1B | Interleukin 1, beta | 0.303333333 |
| 7031 | TFF1 | Trefoil factor 1 | 0.272 |
| 115362 | GBP5 | Guanylate binding protein 5 | 0.264333333 |
| 9770 | RASSF2 | Ras association (RalGDS/AF-6) domain family 2 | 0.262333333 |
| 1917 | EEF1A2 | Eukaryotic translation elongation factor 1 alpha 2 | 0.232 |
| 2788 | GNG7 | Guanine nucleotide binding protein (G protein), gamma 7 | 0.225666667 |
| 3872 | KRT17 | Keratin 17 | 0.22 |
| 3897 | L1CAM | L1 cell adhesion molecule | 0.181333333 |
| 6348 | CCL3 | Chemokine (C-C motif) ligand 3 | 0.173666667 |
| 284217 | LAMA1 | Laminin, alpha 1 | 0.168333333 |
| 64127 | NOD2 | Nucleotide-binding oligomerization domain containing 2 | 0.167333333 |
| 255738 | PCSK9 | Proprotein convertase subtilisin/kexin type 9 | 0.165 |
| 389336 | MGC23985 | Similar to AVLV472 | 0.143 |
| 6659 | SOX4 | SRY (sex determining region Y)-box 4 | 0.127666667 |
| 6319 | SCD | Stearoyl-CoA desaturase (delta-9-desaturase) | 0.118333333 |
| 5137 | PDE1C | Phosphodiesterase 1C, calmodulin-dependent 70kDa | 0.110333333 |
| 5152 | PDE9A | Phosphodiesterase 9A | 0.097333333 |
| 4093 | SMAD9 | SMAD family member 9 | 0.072 |
| 10158 | PDZK1IP1 | PDZK1 interacting protein 1 | 0.070333333 |
| 338398 | TAS2R60 | Taste receptor, type 2, member 60 | 0.0685 |
| 53405 | CLIC5 | Chloride intracellular channel 5 | 0.060333333 |
| 8876 | VNN1 | Vanin 1 | 0.053666667 |
| 3575 | IL7R | Interleukin 7 receptor | 0.052 |
| 6373 | CXCL11 | Chemokine (C-X-C motif) ligand 11 | 0.032 |
| 169611 | OLFML2A | Olfactomedin-like 2A | 0.027666667 |
| 54797 | MED18 | Mediator complex subunit 18 | 0.009 |
| 339501 | MPN2 | Marapsin 2 | -0.002666667 |
| 4879 | NPPB | Natriuretic peptide precursor B | -0.0045 |
| 6659 | SOX4 | SRY (sex determining region Y)-box 4 | -0.023 |
| 126668 | TDRD10 | Tudor domain containing 10 | -0.0265 |
| 6373 | CXCL11 | Chemokine (C-X-C motif) ligand 11 | -0.046 |
| 3625 | INHBB | Inhibin, beta B | -0.06 |
| 85329 | LGALS12 | Lectin, galactoside-binding, soluble, 12 (galectin 12) | -0.079 |
| 3934 | LCN2 | Lipocalin 2 (oncogene 24p3) | -0.106333333 |
| 116372 | LYPD1 | LY6/PLAUR domain containing 1 | -0.139333333 |
| 25840 | METTL7A | Methyltransferase like 7A | -0.151 |
| 28231 | SLCO4A1 | Solute carrier organic anion transporter family, member 4A1 | -0.155333333 |
| 718 | C3 | Complement component 3 | -0.185333333 |
| 8835 | SOCS2 | Suppressor of cytokine signaling 2 | -0.247333333 |
| 28951 | TRIB2 | Tribbles homolog 2 (Drosophila) | -0.252 |
| 5137 | PDE1C | Phosphodiesterase 1C, calmodulin-dependent 70kDa | -0.261 |
| 10076 | PTPRU | Protein tyrosine phosphatase, receptor type, U | -0.268333333 |
| 660 | BMX | BMX non-receptor tyrosine kinase | -0.303 |
| 652 | BMP4 | Bone morphogenetic protein 4 | -0.316 |
| 5209 | PFKFB3 | 6-phosphofructo-2-kinase/fructose-2,6-biphosphatase 3 | -0.333 |
| 4693 | NDP | Norrie disease (pseudoglioma) | -0.361666667 |
| 3625 | INHBB | Inhibin, beta B | -0.367 |
| 9510 | ADAMTS1 | ADAM metallopeptidase with thrombospondin type 1 motif, 1 | -0.368666667 |
| 168002 | DACT2 | Dapper, antagonist of beta-catenin, homolog 2 (Xenopus laevis) | -0.377666667 |
| 84532 | ACSS1 | Acyl-CoA synthetase short-chain family member 1 | -0.397333333 |
| 6659 | SOX4 | SRY (sex determining region Y)-box 4 | -0.43 |
| 3696 | ITGB8 | Integrin, beta 8 | -0.438666667 |
| 3310 | HSPA6 | Heat shock 70kDa protein 6 (HSP70B') | -0.453333333 |
| 9510 | ADAMTS1 | ADAM metallopeptidase with thrombospondin type 1 motif, 1 | -0.468333333 |
| 3570 | IL6R | Interleukin 6 receptor | -0.519 |
| 6947 | TCN1 | Transcobalamin I (vitamin B12 binding protein, R binder family) | -2.587 |
