# Supplementary material for: Suppression of Breast Tumor Growth and Metastasis by an Engineered Transcription Factor
Source: PLoS One. 2011 Sep 13;6(9):e24595. doi: 10.1371/journal.pone.0024595 (PMC3172243; doi:10.1371/journal.pone.0024595)
Supplement: Table S4 — Primers and probes used in this study. (DOCX) [file pone.0024595.s008.docx]

**Table S4. Primer sequences**

| **Gene/miR** | **Probe or**  **Applied Biosystems Ref. #** | **Forward primer** | **Reverse primer** |
| --- | --- | --- | --- |
| **GAPDH** | FAM/MGB #4333764F | - | - |
| **CD52** | Hs00174349_m1* |  |  |
| **END2** | Hs01012714_m1* | - | - |
| **SLC8A2** | Hs00392875_m1* | - | - |
| **CARNS1** | Hs00325412_m1* | - | - |
| **miR-10b** | [002218](https://products.appliedbiosystems.com:443/ab/en/US/adirect/ab?cmd=ABAssayDetailDisplay&assayID=002218&Fs=y&SearchRequest.Common.PageNumber=1&assayType=mirna&chkBatchQueryText=false&srchType=keyword&searchValue=4395329&searchBy=all&msgType=ABmiRNAKeywordResults) | - | - |
| **miR-1** | 002222 | - | - |
| **miR-124** | 4427975 |  |  |
| **miR-34a** | 000426 * | - | - |
| **miR-363** | 001271 | - | - |
| **AATK** | SYBR | AGCAGTGAGGATGAGGACAC | GCCTCCTTGAGCACAAACTC |
| **DACT3** | SYBR | CTCCCCAGCGTCGTCTGCTTTA | ATTCGCTCTCCCCGTAACCC |
| **SOX4** | SYBR | TGCACCCCCAGCAAGA | CACCCCGGAGCCTTCTGT |
| **SRCIN1** | SYBR | CGACTTTGGGCTGGCTCGGCTCA | TGGTCCAGCACCTCGCGGTTCA |
| **GNG4** | SYBR | AGCAGGGGCAGTAGAATGAA | GGATGGGTGTTGGTCTCACT |
