# Supplementary material for: Suppression of Breast Tumor Growth and Metastasis by an Engineered Transcription Factor
Source: PLoS One. 2011 Sep 13;6(9):e24595. doi: 10.1371/journal.pone.0024595 (PMC3172243; doi:10.1371/journal.pone.0024595)
Supplement: Table S5 — Genes differentially regulated with the Maspin cDNA. (DOC) [file pone.0024595.s009.doc]

**Table S5. Antibodies used in this study**

| **Antibody** | **Cat. No** | **Company** | **Dilution and application** |
| --- | --- | --- | --- |
| Maspin (SERPINB5) | 554292 | BD Pharmigen, San Diego, CA, US | WB 1:500 |
| HA-tag | 550513 | BD Pharmigen, San Diego, CA, US | WB 1:5000, IF 1:500 |
| TUBULIN | T5168 | Sigma, St. Louis, MO, US | WB 1:10,000 |
| CD24-PE | 554292 | BD Pharmigen, San Diego, CA, US | FACS 1:10 |
| CD44-FITC | 554292 | BD Pharmigen, San Diego, CA, US | FACS 1:10 |
| Rabbit IgG | 111-035-144 | Jackson ImmunoResearch, PA, US | WB 1:10000 |
| Mouse IgG | 7076 | Cell Signaling, Boston, MA, US | WB 1:5000 |
